# The Power of Wild Plants in Feeding Humanity: A Meta-Analytic Ethnobotanical Approach in the Catalan Linguistic Area

**DOI:** 10.3390/foods10010061

**Published:** 2020-12-29

**Authors:** Airy Gras, Teresa Garnatje, Jon Marín, Montse Parada, Ester Sala, Marc Talavera, Joan Vallès

**Affiliations:** 1 Institut Botànic de Barcelona (IBB, CSIC-Ajuntament de Barcelona), Passeig del Migdia s.n., Parc de Montjuïc, 08038 Barcelona, Catalonia, Spain; tgarnatje@ibb.csic.es; 2 Laboratori de Botànica—Unitat associada CSIC, Facultat de Farmàcia i Ciències de l’Alimentació—Institut de la Biodiversitat IRBio, Universitat de Barcelona, Av. Joan XXIII 27-31, 08028 Barcelona, Catalonia, Spain; jmeirin@gmail.com (J.M.); montse.parada@gmail.com (M.P.); estersalacodina@gmail.com (E.S.); 3 Col·lectiu Eixarcolant, 08700 Igualada, Catalonia, Spain; mtr_27@hotmail.com; 4 Secció de Botànica i Micologia, Facultat de Biologia, Universitat de Barcelona, Av. Diagonal 643, 08028 Barcelona, Catalonia, Spain; 5 Secció de Ciències Biològiques, Institut d’Estudis Catalans, Carrer del Carme 47, 08001 Barcelona, Catalonia, Spain

**Keywords:** Catalan countries, Catalan linguistic area, edible plants, ethnobotany, traditional knowledge, wild food plants

## Abstract

Wild food plants (WFP) have always been present in our kitchen, although they have not always been given the same importance as crops. In the Catalan linguistic area (CLA), covered in this paper, WFP were of great importance as a subsistence food not only during the years of the Spanish civil war (1936–1939) and World War II (1939–1945), but also long before these periods and in the years thereafter. The CLA has been well studied at the level of traditional knowledge on plant biodiversity, and much of this information is collected in a database by the EtnoBioFiC research group. The aim of this work is to carry out a meta-analysis of the WFP dataset of the CLA (only regarding edible uses, drinks excluded) and to identify the most quoted plants, and the information associated with them. With data from 1659 informants, we recorded 10,078 use reports of 291 taxa (278 of which at specific or subspecific levels and 13 only determined at generic level) belonging to 67 families. The most reported taxa, also with highest cultural importance indexes, are *Thymus vulgaris*, *Foeniculum vulgare* subsp. *piperitum*, *Laurus nobilis*, *Rubus ulmifolius* and *Mentha spicata*. The ethnobotanicity index for food plants is 6.62% and the informant consensus factor, also for food uses, is a very high 0.97, supporting the robustness of the information. The results provided and discussed in this work concern a significant part of the edible resources in the territory considered, which is, often and mainly, underestimated and underutilised. Its consideration could be an opportunity to promote closer and more sustainable agriculture. From the state-of-the-art of this question, it is possible to propose old, in some cases forgotten foods that could be newly introduced onto the market, first, but not only, at a local level, which could be interesting for new crop development in the frame of a valorisation of territorial identity.

## 1. Introduction

Wild food plants (WFP) have always been present in the human diet, although in general, and particularly in industrialised areas, they have not always been given the same importance as crops. If Kunkel [1] recorded ca. 13,000 species used in human food (a representative figure, even if it has been increased in the most recent literature), human nourishment depends on slightly less than 100 plant species, around 30 producing the vast majority of what we consume, and only three (*Oryza sativa* L., *Triticum aestivum* L. and *Zea mays* L.) contributing more than half the diet calories [2].

Nevertheless, as a reflection on the long tradition concerning the management of many plants, a non-negligible amount of them is still used, and may have an important significance at a local level. In the Catalan linguistic area (CLA, i.e., the Catalan-speaking regions) studied in this paper the knowledge on WFP has had great importance during the Spanish civil war, World War II, and the years after these wars, as a subsistence or famine food, as also in other places [3], then their use decreased. Similarly, preserved popular knowledge about food plants was useful for subsistence during the siege of Sarajevo and elsewhere in Bosnia and Herzegovina during the war, between 1992 and 1995 [4,5]. Indeed, when the Soviet Union broke up, and Cuba experienced economic difficulties, losing Soviet contributions, traditional agricultural practices regained prominence [6] and ethnobotanical knowledge regarding cultivated and wild plants served to organise food plant supply and significantly alleviated the effect of the crisis [7]. In any case, WFP were also known, appreciated and used before these periods, and the traditional knowledge, which was cumulated not only in emergency episodes, persisted, thus allowing the current use of some taxa even further than at local level [8,9]. In industrialised areas, a couple of generations ago WFP were associated with difficult periods, and acculturation processes have occurred but this has not prevented the continued use of various WFP (e.g., in different places [10,11,12,13,14,15,16,17,18,19]).

In another very different framework, the persistence of traditional knowledge on WFP has been, and is, relevant in the development of high cuisine ([16], and references therein). As a representative example, French (and the World’s) *nouvelle cuisine* would not have been the same (or would not have been) if the Troisgros brothers, Jean and Pierre, had not seen their mother with a handful of *Rumex acetosa* L., and had not been told by her about its use when they were developing an innovative cooking method for salmon [20]. In this respect, in a book titled “*Les recettes de mes grands-mères*” [My grandmothers’ recipes], the Michelin-starred chef Hélène Darroze [21] states “there is an age and an epoch in which we slightly neglect what we received from ours” (cultural erosion, see next paragraph), but “today […] I fall again in the emotion and the recognition of what I learned from mine” (value of traditional knowledge, including that of WFP).

The heritage of ethnobotanical knowledge about food plants, and particularly that of wild plants, semi-domesticated taxa or minor, underutilised or neglected crops is of great relevance in food security and sovereignty, comprising both the right of any person to a sufficient and healthy nutrition and the right of peoples to culturally adequate food [22,23,24,25]. Indeed, food and raw materials have been considered important for food security in different areas of the world, and WFP are important within these raw materials [26]. Ethnoculinary approaches, influencing researchers from different fields in tracing connections between populations often living in different geographic regions, also importantly deal with WFP [27]. WFP are also taken into account in the ever more relevant concept of ecosystem services, as a survival value of cultural ecosystem services, basic for livelihood [28]. In addition, WFP are important as ancestors or relatives of cultivated, including major crop, plants [29], and as a reservoir of genes not present in domesticated taxa or races that could be useful for new crop developments [30]. Irrespective of their relevance, WFP have been the object of cultural erosion [31] and face conservation problems [32], this making their study and possible protection plans (comprising both plants and associated traditional knowledge) highly desirable.

Food composition parameters, basic for the human consumption of any product, are increasingly available for wild edible plants (e.g., [33,34,35,36,37]), suggesting that they could be incorporated into the habitual diet, in some cases even with nutraceutical values [38], in agreement with popular attribution of medicinal properties to many WFP recorded in ethnobotanical prospection ([39] and references therein).The conjunction of traditional knowledge, phytochemical and pharmacological data and cultivation essays resulted in *Reichardia picroides* (L.) Roth, a typical Mediterranean WFP, being proposed as a new health food crop [40], opening a seducing and promising axis in the search for food products.

Ethnobotany of WFP has been intensively addressed in all continents with a permanent population since the classical times of modern ethnobotanical research (e.g., among numerous papers [10,11,12,14,15,17,19,41,42]). The CLA is among the best-studied areas in Europe at the level of traditional knowledge on plant biodiversity ([43] and references therein), and much of this information is collected in a database of our research group. This database currently contains information of 3039 informants and 1778 plant taxa. This represents a total of 118,537 use reports (UR), of which 69,170 (58.35%) correspond to medicinal uses, 31,415 (26.50%) to food (including beverages) uses, and 17,952 (15.15%) to other uses. Several ethnofloristic or review papers focused on food plants have been published from this cultural area [13,44,45,46] as well as more general papers containing information on WFP [18,47,48] or dealing with reasons for the use of WFP [9], but to date no attempts to carry out a global or at least major analysis has been performed. Indeed, meta-analytical work on WFP in large areas is, as far as we can see, lacking elsewhere.

In accordance with the reasons stated in the last paragraph, the aim of this paper is to present a meta-analysis of a significant part of the WFP dataset of the Catalan-speaking territories, inventorying the plants and the information associated with them, and identifying the most relevant ones. With this work we intend (a) to build a catalogue of Catalan-culture WFP, as in other ethnic groups often underestimated and underutilised, (b) to analyse data recorded from different points of view in order to pave the way for a meta-analysis on this subject that could help to envisage a further comparative study at higher levels (European, Mediterranean), (c) to identify, according to the ethnobotanical information, the WFP that could have the added value of being considered as nutraceuticals, and (d) to indicate, suggest or propose old, in some cases forgotten traditional foods that could be newly introduced onto the market, first -but not only- locally, or that could be of interest for new agronomic and commercial possibilities.

## 2. Methodology

### 2.1. Study Area

**Figure 1:**
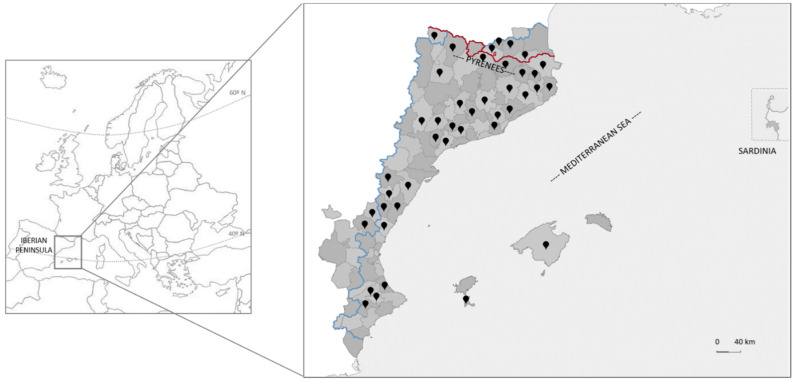
Territories studied (black drops) in the Catalan linguistic area and its location in Europe. The blue line defines the current zones where Catalan language is spoken and the red line the borders between states. The small subdivisions in the continental part correspond to administrative districts (called ‘*comarca’*, pl. ‘*comarques’*, in Catalan).

The CLA, often also called Catalan-speaking or Catalan-language territories or Catalan Countries, constitute a unit that has been considered and well studied from geographic [49], physiographic [50], floristic [51,52], vegetation [53], linguistic and cultural [54], and ethnoculinary and gastronomic [55,56] points of view. It occupies the eastern part of the Iberian Peninsula, including a northern Pyrenean portion, the Balearic Islands and the city of l’Alguer on the island of Sardinia (Figure 1). Politically, it is currently made up of four states, Andorra (all the territory), France (Northern Catalonia or Eastern Pyrenees department), Italy (l’Alguer), and Spain (Balearic Islands, Carxe -a small area in Murcia-, Catalonia, a portion of eastern Aragon, and Valencia). Its extension is ca. 70,000 km^2^ [52] and it is inhabited by around 14,000,000 people [57].

From the Mediterranean Sea level to 3143 m a.s.l. in Pica d’Estats (Pyrenees), the landscape of the area considered is structured in several parts with distinct floristic and vegetation traits [52,53]. The main part, at the lowest altitudes, belongs to the Mediterranean region, with *Quercus ilex* L. and derived communities, complemented with *Quercus coccifera* L. and *Thymus vulgaris* L. communities in warm and dry places. Low and median mountain parts are dominated by deciduous *Quercus* and *Fagus sylvatica* L. communities, with *Pinus sylvestris* L. High mountain areas in the southern, more arid zones present landscapes with *Pinus sylvestris* and *Juniperus sabina* L. In the Pyrenees, the dominant trees of the high mountain areas are *Abies alba* Mill. and *Pinus mugo* Turra subsp. *uncinata* (Ramond ex DC.) Domin, with alpine meadows and a small snow level being found at the highest altitudes. The approximate number of plant taxa (including species and subspecies) in these territories is 4300 autochthonous plus 1200 allochthonous [58].

Even though it includes several big or very big cities (in decreasing order of inhabitants: Barcelona, Valencia, Palma, Perpignan), and not ignoring the importance of industry and tourism to its economy, the area studied still has a relevant rural component, and agriculture and natural spaces (including reserves, parks and other protected zones) are important. Catalan is the common language to the whole area, with two main dialects (eastern -including, among others, Balearic subdialects- and western -including, among others, Valencian subdialects-) [59] and it has official, co-official or a protected status in the different places. Phytonymy in Catalan language is quite rich, with ca. 35,000 Catalan plant names recorded for around 6000 taxa [60], indicating a rather intense contact between humans and plants in the area. Apart from this, French, Italian, Occitan, Sardinian and Spanish languages are also spoken and have different degrees of official status in one or another of the zones. The area considered includes the places where Catalan language is spoken, was spoken in the past (such as Oriola/Orihuela, where it was spoken at least until 17th century and has left an interesting substrate) or was not spoken but, by geopolitical reasons, it has had some influence (as current Catalan-speaking areas receive influences from neighbouring languages), in some cases reflected on folk plant names, such as in a few Spanish-speaking districts in Valencian community and a few Occitan-speaking areas in Spanish and French CLA. In addition, it is remarkable that, in the biggest city of this area, Barcelona, alone, more than 300 languages are spoken, due to migration flows [61]. In some cases, this is associated with the use of specific food plants (usually cultivated or even freshly imported ones) by some linguistic and cultural groups (e.g., in a city bordering Barcelona, [62,63]).

### 2.2. Databasing and Data Selection

Traditional knowledge on plant biodiversity has been collected by ethnobotanical prospection in the studied area in modern times from the pioneering work of Pius Font i Quer [64] and more recently, and in a continued manner to date, from the first academic works of Mulet [65] and Muntané [66]; see a review in Vallès [42]. The information has been collected through semi-structured ethnobotanical interviews (see details in [18] and references therein) and included in a database by our research group (www.etnobiofic.cat). The meta-analytic work performed in the present paper covers 31 research studies belonging to different regions throughout the CLA (Figure 1, Supplementary Material S1). They constitute a representative set from both a geographic and a dialectal point of view: Catalan language is divided in two main dialects, western and eastern [59]. As first-level subdialects, within western we find north-western and Valencian, both represented, and within Eastern, apart from Algherese -the only one not represented, among other aspects because the current pandemic made impossible a first prospection that was foreseen-, we find northern, central and Balearic, all included in the study. Indeed, within Valencian, Central and Balearic, some second-level subdialects are also represented.

The information concerning WFP has been recovered from the mentioned database. A minor bias exists, because in one out of the 31 studies included in the dataset [65] each taxon is assigned to a municipality instead of to an informant, as is the case in all the other works. This can result in a slight underestimation of use reports and also of the indexes that include them. As WFP, we consider in this work wild vascular plants used for food purposes. Only edible uses have been taken into account, and a work is in progress on beverage uses (Gras et al., in prep.). Neither fungi and non-vascular plants, including algae, nor cultivated plants are considered. In both cases, interesting results for further papers exist; in the last one particularly with attention to local, neglected or underutilised minor crops. Under the term ‘wild’ we include strictly wild autochthonous taxa as well as some introduced or naturalised taxa recorded in the manual flora of the Catalan Countries [52].

For taxa nomenclature we follow Bolòs et al. [52], a flora specifically covering the area considered, and for family attribution we follow APG IV, the last Angiosperm Phylogeny Group’s arrangement to date [67].

### 2.3. Data Analyses

We calculated the ethnobotanicity index (EI; [68]), the quotient between the number of plants used (here taking into account WFP) and the total number of plants that constitute the flora of the territory (authochthonous plants, see an estimation above), expressed as a percentage, in order to have a general idea of the relevance of WFP in the area considered. We also calculated the informant consensus factor (F_IC_; [69]), the ratio of the number of UR minus the number of used taxa to the number of UR minus one, in order to assess the consistency or robustness of the traditional knowledge of WFP in the territory. The cultural importance index (CI; [70]), the sum of the proportion of informants that mention each species use, has also been calculated to identify the plants most valued by the informants.The descriptive statistics were carried out with Excel (Office 2008, Microsoft Ibérica, Pozuelo de Alarcón, Spain. The relationship between the preparation form and the most quoted plants was visualized by an alluvial diagram using RAWGraphs [71].

### 2.4. Complementary Interviews

In addition to the analysis of the results compiled in the database as mentioned, we performed brief, directed ethnobotanical interviews performed in September 2020 on nine people in several areas covered by this study to obtain only qualitative information on aspects related to the appreciation of some particular plants or on WFP going beyond the local use, even including current use in restaurants (see Subsector 3.6 in the Results and Discussion section). The informants were selected following our experience in this field, just to have complementary information. On the one hand, two interviewees were, respectively, the brother and the son of an informant -already deceased- present in the database analysed. On the other hand, seven interviewees were more or less connoisseurs of current wild plant appreciation, commerce or use in markets or restaurants. The information provided by these people and used here is explicitly indicated in Section 3.8 (in one case in Section 3.2) and all of them are summarised in Supplementary Material S2. We followed the ethical principles of the International Society of Ethnobiology [72], the interviewees gave their oral informed consent prior to the conversations, and the interviews were developed in the Catalan language, common to interviewers and interviewees.

## 3. Results and Discussion

The pool of WFP traditionally used for food—excluding beverages—in the Catalan linguistic area is constituted by 291 taxa, 278 at specific and subspecific level and 13 determined only at generic level, belonging to 193 genera and to 67 families, according to the information gathered in ethnobotanical prospection from 1659 informants (53.65% men, 46.35% women). The total number of UR is 10,078. Data on scientific names, part of plant used and preparation are presented in Supplementary Materials S3 and S4. 

The number of WFP recorded can be considered high, when it is compared with those reported from other zones, which in most cases comprise drinks apart from foods, which are the only products considered in this paper: Spain (419; [73]), several areas of Greece, Italy and Spain (318 in total, 147, 84 and 173 in each country; [74]), North West Iberian Peninsula (97; [75]), Northern American native peoples (103; [41]), in Karelia (70; [19]), several Turkish areas (27—in a small island—, 61, 86, 154; [76,77,78,79] or West Sumatra (106; [80]). We quoted here works dealing with socioeconomically and geographically different areas, including the one our studied zone belongs to. We are aware that the number of plants used may depend on the number of plants of the local flora and on the sampling effort in each case; this is why we considered works from quite different places, to show variability and give at least an approximate idea. In a review of 64 Mediterranean and neighbouring areas, the biogeographical region, which our studied area belongs to, Rivera et al. [81] recorded between 14 and 565 (mean 133.11) wild food taxa, including fungi and considering drinks, again raising the number of taxa recorded in this paper to a high level.

### 3.1. Quantitative Ethnobotany

The ethnobotanicity index (EI), considering the taxa at the specific and subspecific level, and considering for the total number of taxa present in the flora the above-mentioned estimation of 4200 [58], is 6.62%. This is the percentage of the total autochthonous flora in the territory studied with traditional food (drink excluded) use. It is slightly lower than the 8.90% reported for one of the areas here analysed [13], which is logical because in the latter case the flora is much more restricted and, in addition, beverages are taken into account. In Spain, Tardío et al. [73] report 419 WFP restricted to edible use, as in the present paper, and Tardío and Pardo-de-Santayana [82], 464 WFP, including those used in beverages. Using the number of species and subspecies of Iberian, Balearic and Canarian flora (8882) provided by Pando et al. [83] minus 136 taxa endemic to continental Portugal [84], the EI for WFP in Spain as a whole is 4.79% (edible plants) or 5.31% (all food plants, including drinks), lower than the one calculated for the Catalan linguistic area for edible uses only.

Out of the 278 specific and infraspecific taxa recorded, 157 (56.47%) fit the reliability requisites indicated by Le Grand and Wondergem [85] and Johns et al. [86] of having three or more reports from independent informants. The number and proportion of taxa that would be the most indicated for further food development processes is high, encompassing clearly more than half the WFP recorded. Of course, plants with less than three user reports should not be neglected, since their low reports could be either the remnants of an earlier wider knowledge or the product of local cultural specificities and preferences, even in a globalised world. This is why all recorded WFP are presented in Supplementary Materials S3 and S4.

The informant consensus factor (F_IC_), calculated for the 278 taxa determined at specific and subspecific levels, has a value of 0.97, in a scale where the maximum is 1.00. It is slightly higher than the 0.92 reported for one of the areas here analysed [16], and it is placed among the highest in different territories where this index has been calculated (Ref [47], and references therein). This fact strongly reinforces the above-mentioned reliability requisites assessed in terms of a minimum number of UR. This index was created as “a new approach for identifying potentially effective medicinal plants”, and it can also be used for the same purpose regarding food or other kinds of useful plants. A solid consensus (and not a dispersion of uses with very scarce reports) among informants in the use of a pool of WFP for a purpose (in the present case, food) indicates a trial and error process, and the subsequent agreement of people as concerning some plants with a kind of use rather than random individual uses. This accounts for the robustness and reliability of the useful plants claimed, this being an important factor to identify plants that could be used as food in the future beyond the local level.

Apart from the two evidences of plant-use reliability (three or more independent quotations and F_IC_), there is another indication of the appreciation that the informants have for the plants they use: the cultural importance (CI) index. Its values are highest in the first plants of the UR ranking (see more comments in Section 3.2), the maximum being 0.52 for *Thymus vulgaris*. This index, combined with the two commented earlier, could be of good value when selecting WFP that would be positively received in local markets.

### 3.2. Most Used Taxa at Family, Genus and Species Levels: Facts and Reasons

**Figure 2:**
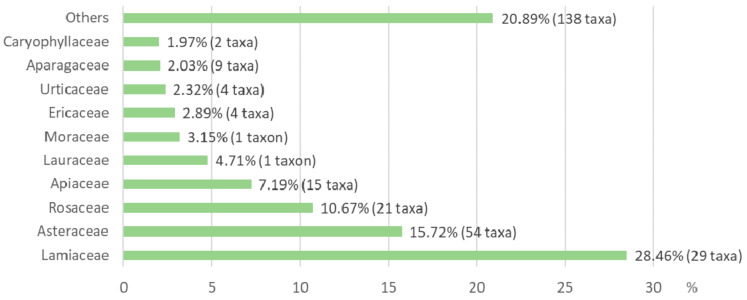
Most reported families, with number of taxa per family, of wild food plants in the area studied.

The five most quoted ones are Lamiaceae (29 taxa, 28.46% of UR), Asteraceae (54, 15.72%), Rosaceae (21, 10.67%), Apiaceae (15, 7.19%) and Lauraceae (one, 4.71%), the first three encompassing more than half of the total UR (Figure 2). All but the last one are large or very large families, in terms of number of taxa, have a wide distribution, are among the most common in the Mediterranean region [52], and appear as leading families in most ethnobotanical prospections in this area, either regarding food or medicinal uses ([48,75] and references therein). In addition, all but the last one are among the five first families in the present work in number of taxa used. The outstanding presence of Lamiaceae in the first place, with more than one quarter of the total UR, is explained by the richness of this family in essential oils, which makes it particularly suitable as spice or condiment. In its turn, the Lauraceae are not a very big family and have only one autochthonous species in the territories studied (*Laurus nobilis* L.), but its importance as a condiment makes it highly valued by the informants and present in almost all houses.

The families numerous in taxa and widely distributed, as those here mentioned, are good candidates for leading the ranking of more traditionally known, and used not only for these rather evident reasons, but also for taxonomically-directed processes inducing people to choose useful plants. Saslis-Lagoudakis et al. [87] found close phylogenetic relationships between taxa used for the same medicinal purposes in three distant ethnofloras. Concepts such as ethnobotanical convergence [88,89,90] or utilitarian equivalence [91] insist, with different nuances, on the relationship between taxonomical affinities and plant use. Most of the examples from the works dealing with this subject refer to traditional medicinal uses, but the idea perfectly applies to food plants, and this idea can even be extended to the genus level. Indeed, Garnatje et al. [89] mention the case of two *Origanum* (Lamiaceae) species used as the condiment of the same kind of food preparation in the Western and Eastern parts of the Mediterranean region, and of food consumption of *Cucurbita pepo* (Cucurbitaceae) flowers in Mesoamerica and South-West Europe. Thus, the territories studied in the present paper, less distant that in the examples quoted, and sharing language and culture, but with important different ecological conditions along the area, make good candidates for processes of taxonomically-oriented plant selection. Chemical composition, close in phylogenetically-related taxa, is claimed as responsible for this phenomenon in medicinal uses ([91] and references therein), and the same can be stated for food utilisation, even more so taking into account the fact that many food plants also have medicinal uses, as we will comment later. Related with chemical composition and also sensitive to phylogenetic closeness, sensorial profiles are relevant for the acceptance of edible plants by people [92,93].

Out of the 193 genera, the first 10 represent not far from 50% of the UR. Nine out of those 10 genera belong to the five most reported above-quoted families. The first one is *Thymus*, with a few species, the most relevant among which, *T. vulgaris*, will be commented on later. *Laurus* appears in the fifth position, with 4.71% of the UR. The only top ten taxon not belonging to the most quoted families is *Ficus carica* L. (Moraceae), the fruits of which are much appreciated and eaten either fresh, dried or in jam.

As for the species, the most frequently used one is *Thymus vulgaris*, not far from 10% of the total food plant UR (Table 1). The statement of one informant “*n’hi ha a totes les cases*” (‘it is present in every house’) [94,95] is a clear description of the situation, well complemented with the following assertion: “*jo esmorzo cada dia d’un plat de sopa de pa i frigola*” (‘I have for breakfast every day a dish of bread and thyme soup’) [96]. The predominancy of *Thymus vulgaris* is very common in Mediterranean ethnobotanical research ([18,48] and references therein). The top five plants account for more than one quarter of the total UR and the 30 first ones, near three quarters of them (respectively 27.85% and 73.57%, Table 1). The five first plants in the ranking, the only ones with more than 4% of the total UR, belong to the top five genera and to four out of the top five families, thus reinforcing their relevance in the territory considered. The first-classed taxa exhibit the highest CI index values (Table 1), which adds a cultural reason to the frequency of citation. To exemplify this, we need only to refer to an ethnobotanical survey in a geographically not very distant area, located in northern Iberian Peninsula, but belonging to another cultural group [70], the plant ranking is quite different and, particularly, the only coincidence in both areas within the top 20 taxa (*Origanum vulgare* L.) has there a CI index of 0.42 whereas in our study it has a value of 0.20.

Of the 30 taxa first classed in the ranking, there are many with current uses, despite an apparent acculturation, or traditional knowledge erosion, which takes place in industrialised zones. Even if in more of the studies occupying the present meta-analysis there are not quantitative appreciations of persistency of uses, our experience in ethnobotanical prospection allows us to comment on a few situations with the plants that occupy the first positions in the list. First, *Thymus vulgaris*, *Foeniculum vulgare* Mill. subsp. *piperitum* (Ucria) Cout., *Laurus nobilis*, *Rubus ulmifolius* Schott and *Mentha spicata* L., the top five taxa, are nowadays frequently used in many homes throughout the territory. Conversely, there is a pool of plants that are very well known and remembered by people but that have fallen into absolute disuse or that are very rarely used. This is the case of a set of plants that used to be employed as salads and which, since the ease of availability of lettuce and related plants in the markets, are now seldom used. Some plants of the lettuce family (in decreasing order of UR, *Reichardia picroides*, *Chondrilla juncea* L., *Taraxacum officinale* Weber in Wiggers and *Cichorium intybus* L.), and some other taxa (*Papaver rhoeas* L. and *Portulaca oleracea* L.) are in this situation. The description recorded for this change in use is clear: “*ara no cal anar a buscar aquestes misèries*” (‘now there is no need to look for these lowly plants’) [97], but see later some nuances in Section 3.6. Finally, there are, in the first 20 positions, some other plants that continue to be in rather frequent use, in a similar way to those commented on in the top five taxa, e.g., *Asparagus acutifolius* L., *Origanum vulgare*, *Rosmarinus officinalis* L. and *Satureja montana* L. It is worth mentioning that in one case, *Origanum vulgare*, the modern, recent use complements -and probably surpasses- the traditional one, as perfectly reasoned in this statement, “*s’usa per a preparar patates a la cassola, guisats, guisats de pollastre, conill i en el cuinat de caragols, però també com a aromatitzant de la pizza*” (‘it is used to prepare potatoes in the casserole, stews, chicken stews, rabbit, and in cooking snails, but also as a flavouring for pizza’) [98], which mentions first what is probably the most common use.

Apart from being commonly distributed in general, the ease of availability, one of the reasons for traditional plant knowledge and collection, is given in terms of the proximity of plants to the place where people live [86], i.e., the floristic composition of the local landscape [99]. This is determinant for plant choosing, as we have verified in many punctual ethnofloristic prospections (e.g., [16]) and we confirm once again in the present meta-analytic work. The informants remark that many plantsthey were or are collected not far from home: expressions similar to “*creix pels marges*” (‘it grows by the sides’) [96,97], meaning that the plant is found in small slopes or path sides close to home, is almost always pronounced by the informants, for instance, for one of the top ten plants, *Reichardia picroides*. For *Papaver rhoeas*, “*n’hi ha molta*” (‘there is a lot of it’) [100] is stated, indicating that it is very easy to obtain it. Apart from this, some of the highly appreciated WFP plants were cultivated (often originally collected in the wild, in some cases bought) near the house to ensure ease of collection, such as the above-mentioned *Ficus carica* and *Laurus nobilis*.

Not only most reported plants are interesting and significant. Scarcely-quoted plants are also important, since in many cases they are representative of particular geographical zones and, in addition, very often they incorporate elements of local identity. This is the case, for instance, of *Taraxacum dissectum* (Ledeb.) Ledeb. in high mountain regions of the Pyrenees [16,66], of *Cytinus hypocistis* (L.) L. and *Pyrus spinosa* Forssk. in arid inner areas of Catalonia [18], of *Salicornia patula* Duval-Jouve in littoral areas (Marín et al., unpubl. res.) and of *Origanum virens* Hoffms. et Link and *Thymus piperella* L. in southern Valencian areas (Supplementary Material S2).

Good taste and healthy (most probably including, first of all, nutritive) properties are also important for people when making the decision to use a taxon as food. In a study focused on 21 WFP species in three of the territories considered in the present work, the appreciation of the flavour of a plant was determinant for its use, whereas a disgusting taste was a relevant motivation to stop eating a WFP [9]. Expressions from our informants like “*la farigola és molt bona*” (‘thyme is very good’) [97], “*fa molt bona olor quan està florit*” (‘it smells very good when in flower’, concerning *Sambucus nigra* L.) [101], “*la truita d’espàrrecs té gust!*” (the asparagus omelette is tasty!’) [95], “*m’agrada molt, té molt bon gust*” (‘I like it very much, it is very tasty’, for *Papaver rhoeas*) [101] or “*són boníssimes, nosaltres sempre que en trobem n’agafem*” (‘they are extremely good, we collect them whenever we find them’, referring to *Rubus ulmifolius* fruit) [101] account for the importance of appreciating the organoleptic properties of WFP. The quoted sentences are qualitative appreciations given by informants. Recently, Talavera ([97] and work in progress) started quantitative tests on a high number of informants (over several years) who are asked to taste different preparations with WFP and give responses to different questions on their smell, taste and other characteristics.

Finally, we can also consider an economic motivation for WFP collection or consumption. The informant’s statement “*en van a collir de silvestres per a vendre’ls a mercat*” (‘someone is collecting wild ones to sell them in the market’) [96], referred to *Asparagus acutifolius*, and is quite clear and will be discussed in Section 3.6. “*En veníem, com les maduixes de bosc*” (‘we used to sell them, as well as wild strawberries’ [102], referred to the fruit of *Rubus idaeus* L., implying also that of *Fragaria vesca* L.), also exemplifies, as in other similar cases, the possible economic revenues resulting from WFP collection. Speaking of *Taraxacum dissectum*, one informant remembered that “*al temps de la misèria passaven dones que n’havien anat a collir a la muntanya i les venien, un plat valia 10 cèntims*” (‘in times of scarcity, some women passed after collecting the plants in the mountain and sold them; a dish cost 10 cents’) [47]. For *Rubus idaeus*, informants also quote “*la melmelada de gerds és la més bona*” (‘red raspberry jam is the best one”) [103], indicating they also use them, i.e., both taste and economy causes converge in the use of this WFP. The economic factor is not only the income perceived by selling plants, but can also be considered in terms of saving by using WFP: If, for instance, traditional knowledge allows people to collect and to prepare jams or similar products from *Arbutus unedo* L., *Rubus idaeus*, *Rubus ulmifolius* or *Vaccinium myrtillus* L. picked by themselves in the field, they should not therefore have to buy the same products in the supermarket.

### 3.3. Parts of Plants Used

**Figure 3:**
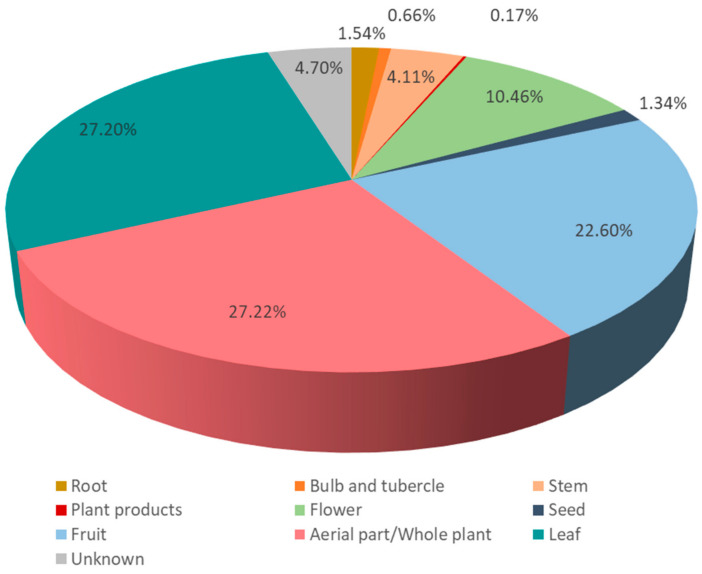
Parts of the wild food plants used in the area studied.

The parts of the plants used for food purposes are presented in Figure 3. A practically absolute predominance of aerial parts is observed: the stem, the leaf, the complete aerial part (or the whole plant, the distinction between both categories being sometimes difficult), the fruit (including its parts, fructification and infructescence), and the flower (comprising its parts, inflorescence and floral summit) altogether represent 91.59% of the total UR. In contrast, subterranean parts are a small minority: root, tuber, bulb and rhizome encompass 2.20% of the total UR. Plant products (latex, nectar or sap) cover only 0.17% of the total UR. Finally, a 4.70% of UR correspond to parts not reported (most often ignored or forgotten) by the informants. The clear prevalence of aerial parts and minority subterranean ones is shown also, for instance, in research performed in the eastern Mediterranean region [76,77], northern Europe [19] and eastern Asia [80]. This consistency in quite geographically, culturally and socioeconomically different regions suggests a common pattern in parts of WFP used. This pattern is not intuitive, since everyone has in mind the relevance of subterranean parts in cultivated food plants, but it could be interpreted as an adaptation of the idea of Johns et al. [86] that the wild plants growing near the human settlements are among the most used. In this case the ease of accessibility does not regard the plants themselves, but their most apparent parts, which are aerial ones. The fact that the underground parts have to be harvested mainly in the plants’ vegetative state, making it difficult in some cases to undoubtedly identify the taxa, could have some influence on the situation described. In any case, a beneficial side effect may be mentioned, since, as stated by Pawera et al. [80], an intensive harvest of subterranean parts of WFP could be harmful in terms of plant conservation issues. Apart from this, a lower palatability of subterranean parts in respect to other parts of plants could be an explanation for their scarce use [97].

### 3.4. Preparation Forms

**Figure 4:**
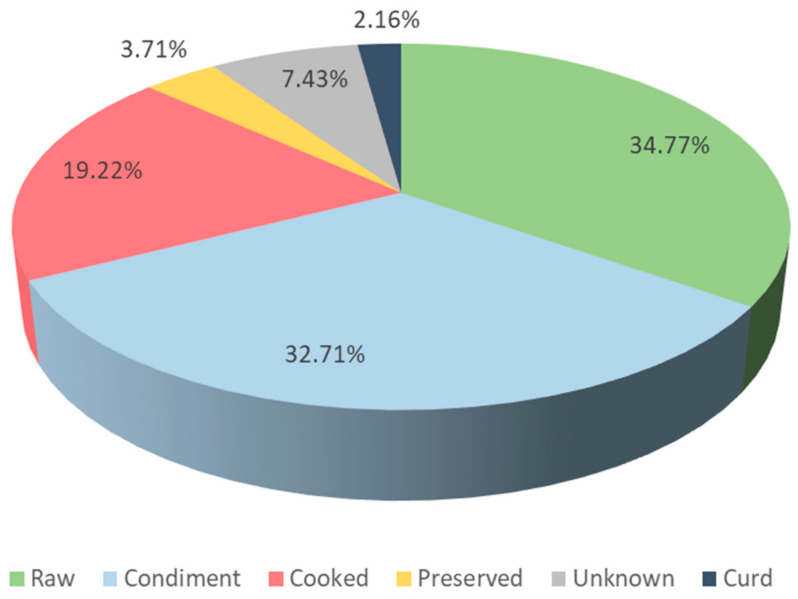
Modes of preparation of wild food plants in the area studied.

**Figure 5:**
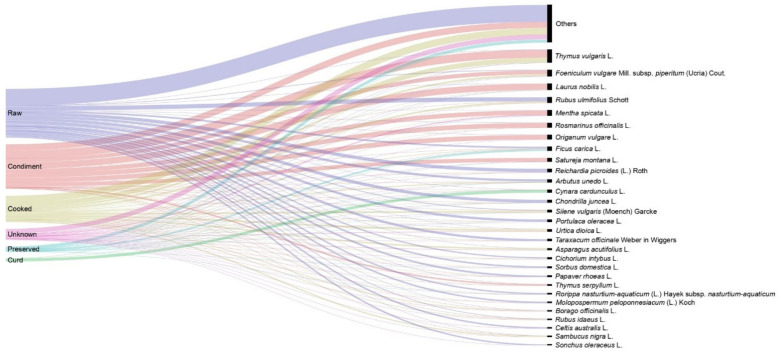
Modes of preparation of the most reported wild food plants in the area studied.

The types of preparation of the WFP are shown in Figure 4 and Figure 5. In Table 2, 10 kinds of traditional foods elaborated with WFP are presented, with an explanation of their preparation and some examples of plants concerned in each type of food. WFP in the Catalan linguistic area are, in decreasing order, either ingested raw (34.77% of the total UR), used as a condiment (32.71%), ingested cooked (19.22%) or used preserved (3.71%). There is a 7.43% of the UR for which no information of the preparation type is recorded, and a 2.16% of the UR corresponding to the utilisation of a plant as curd. Among these types, the plants that are ingested in high portions are those more representative in feeding terms, even if condiments also represent interesting nuances in this respect.

The simplest procedure of preparation largely dominates, since the use of fresh plant material (implying just cleaning) and the use as a condiment (implying only cleaning and in most cases drying) represent, according to the percentages quoted in the last paragraph, around one third each of the total UR. This may have at least three explanations. First, the importance of salad WFP, in agreement to that of salads with cultivated plants, very typical in every diet. Nevertheless, other preparations than salad exist for fresh WFP, such as the ingestion of *Malva sylvestris* L. or *Rubus ulmifolius* fruits as aperitif and dessert, respectively. Second, the relevance of spices and condiments, again in every diet, and the large number of either cultivated or wild plants, mostly with essential oils, with this function. Finally, the ease of use itself. Preparing (exceptionally or regularly) a WFP salad is simple, and seasoning a pizza, a stew or a salad or flavouring an oil with a condiment, too. This last point parallels the fact that tisanes prepared by a simple infusion or decoction are indicated among the most important pharmaceutical forms of many ethnobotanical studies centred around medicinal plant uses ([18] and references therein). In any case, simplicity in use does not necessarily imply simple or vulgar dishes. Many condiments are very relevant in cooking or preparing plants, such as *Satureja montana* and *Thymus vulgaris* for olive treatment and preservation all along the area studied, or *Thymus piperella* for the same purpose, to cook snails and to prepare ‘*gaspatxo’* -a traditional SE Iberian solid dish, different from the cold soup called ‘*gaspatxo’* in Catalan and ‘*gazpacho’* in Spanish- and *Origanum virens* for cooking typical fish or veal stews, the latter two species in southern Valencian territory. It is worth mentioning that attempts at home cultivation are not at all rare among the informants, at a local level in the homegardens or even in pots on balconies, of some condiment WFP they particularly appreciate and use frequently, as often stated during the interviews, with sentences such as “*aquí al jardí en tenim de plantada*” (‘we have it planted here in the garden’ [96,103], in this case referring to *Thymus vulgaris*).

Almost one quarter of the total UR belongs to the use of plants cooked or preserved. This is a minority but representative proportion of more complex preparation forms, revealing that not because we are dealing with WFP should their preparation always be reduced to almost nothing. In fact, most of the methods employed in the kitchen for cultivated plants are applicable to WFP, as we shall show with a few examples.

**Figure 6:**
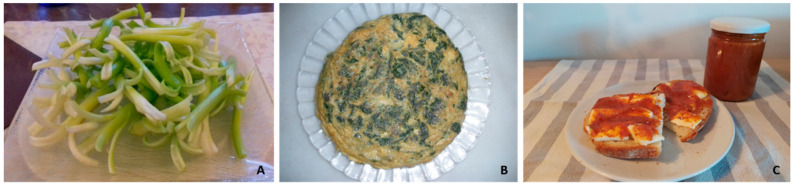
Examples of wild food plants prepared for consumption. (**A**) *Molopospermum peloponnesiacum* in salad; (**B**) *Chenopodium bonus-henricus* in omelette; (**C**) *Arbutus unedo* preserved in jam.

Regarding cooked plants, they may be boiled in water (such as *Silene vulgaris*) or in milk (*Chenopodium bonus-henricus* L.; this was commonly done by the shepherds, who then ate the plant and drank the aromatised milk), fried in oil (*Sambucus nigra*), prepared in omelette (*Asparagus acutifolius*, *Chenopodium bonus-henricus*, *Malva sylvestris*, *Urtica dioica* L.) (Figure 6), stewed, usually in meat dishes (*Castanea sativa* Mill.), prepared in a kind of cake called ‘*coca’* (*Silene vulgaris*) or in other sweet (*Papaver rhoeas*) or salted (*Silene vulgaris* in Mallorca’s traditional salty pie ‘*cocorroi’*) cakes, or as a soup (*Chenopodium bonus-henricus*, *Mentha* sp., *Thymus vulgaris*). In many cases, WFP, in any of the forms of preparation, are cooked and consumed together with other plants, usually cultivated, such as boiled *Urtica dioica* accompanying *Solanum tuberosum* L. or the same WFP species as an ingredient of a soup or even of cannelloni. In Valencian territories, Pellicer [104] indicated that *Asparagus acutifolius* may be a component of a traditional Valencian dish called “*borreta*”, together with *Allium sativum* L., *Beta vulgaris* L., *Phaseolus vulgaris* L., *Solanum tuberosum*, *Spinacia oleracea* L., apart from codfish and eggs. Similarly, in the Pyrenees, *Chenopodium bonus-henricus* may be added to a very popular dish, “*trinxat*”, based on mashed *Brassica oleracea* L. and *Solanum tuberosum*, with pork bacon.

As in the case of cultivated plants, WFP preserves may be prepared salty or sweet. Many berries and other fruits (such as *Arbutus unedo*, *Rosa canina* L., *Rubus idaeus*, *R. ulmifolius* or *Vaccinium myrtillus*) (Figure 6) are boiled with sugar, and the jam or similar products obtained are consumed for breakfast or as a dessert, often spread on bread or cakes. Since these fruits are boiled, we considered them, as cooked, as a preparation form, even if they constitute a preserve. As for salty, *Crithmum maritimum* L. leaves are pickled with wine vinegar and salt, and used to accompany olives, bread spread with tomato and olive oil, ‘*trempó*’ (Balearic salad) and ‘*sopes*’ (a traditional kind of soup typical in Mallorca). Again, simple uses together with more complex ones are found, following a similar model to the one of cultivated plants.

A small number of reports, representing only 2.16% of the total, correspond to plants used to curdle milk and prepare cheese and related dairy products. This use, comparable with that of industrial rennet of animal or other origins, is mostly centred on *Cynara cardunculus* L. flowers, separated from the inflorescence and added, directly or its maceration water, to the milk when it starts boiling or when it is warm but still far from boiling, depending on the places.

### 3.5. A First Step towards the Corpus of the Rarely Reported WFP in the CLA

To evaluate the range of the use as food of the taxa analysed in this study, we checked our information with a comprehensive repository of useful plants, comprising WFP, as is the Plants for a Future database (PFAF) (https://pfaf.org). This validation is a first approach to detect the rarely reported plants.

Out of the 278 WFP taxa recorded in this paper, 218 (78.42%) appear in the PFAF database as edible. Some of these WFP are considered with a high edibility rating, such as *Rumex acetosa*, whereas some others, such as *Cytinus hypocistis* have attributed a low edibility rating. This means that most WFP employed in the CLA are more or less common and well known as edible. Nonetheless, a non-negligible pool of plants, more than one fifth of the total, does not appear in this database. Some of them are widely distributed and for sure used in other regions, such as *Apium nodiflorum* (L.) Lag. subsp. *nodiflorum*, but some others are rarer.

A number of the 60 taxa (21.58%) not present in the PFAF database are importantly reported in the CLA and, although they are not endemic, most of them are not so common elsewhere. We provide here a few examples of different territories. *Molopospermum peloponnesiacum* (L.) Koch, *Saxifraga aquatica* Lap. and *Taraxacum dissectum* are common in the Pyrenees and actively searched during a specific time of the year. In inland, dry territories *Pyrus spinosa* constitutes a similar case, as well as *Thymbra capitata* (L.) Cav. in the Balearic Islands and *Thymus piperella* in the south Valencian area. A much more in-depth revision, including almost all European literature should be undertaken in order to definitely establish a pool of WFP particularly representative of the CLA, but now a not inconsiderable first approach is done.

### 3.6. Links between Nourishment and Medicine: WFP as Folk Functional Foods

Many food plants (including WFP) have been reported to also have medicinal uses, entering within the concept of nutraceutical [81,105]. *Thymus vulgaris*, the top plant in terms of UR and CI in this study, provides a clear example of what we called folk functional food [39]. This taxon is claimed to possess many food (Table 1 and Supplementary Material S3) and medicinal ([18] and references therein) properties. One of the most typical preparation forms for food use is a soup made with water or a broth, bread, olive oil and the plant, sometimes complemented with *Allium sativum* and eggs. This soup is reported to have medicinal properties, and in some cases it is indicated that it was prepared for cold or digestive problems, and that it reinforces the organism: *“la farigola és molt bona; els pastors se’n feien, de sopa de farigola, per això estaven tan forts*” (‘thyme is very good; shepherds prepared thyme soup every morning, this is why they were so strong’) [106].

Out of the 278 taxa here recorded, 39 (14.03%) appear in the comprehensive dictionary of nutraceutical and functional food [105], indicating that a non-negligible amount of WFP has medicinal properties that make these plants, apart from nutritious, particularly salutary, and confirming the role of ethnobotany in healthy eating [107]. Some examples cited as nutraceuticals and present among the most cited plants (see Table 1) in our territory are *Thymus vulgaris*, *Foeniculum vulgare* subsp *piperitum*, *Mentha spicata*, *Rosmarinus officinalis*, *Origanum vulgare*, *Satureja montana*, *Urtica dioica*, *Taraxacum officinale* and *Asparagus acutifolius*. The number of nutraceutical plants would increase taking into account recent research, complementing the mentioned dictionary. For instance, see the information on this subject regarding *Portulaca oleracea*, not included in the dictionary, in the section on markets and industrialised products of Section 3.6. Just to give a few more examples of plants not present in the dictionary, Guijarro-Real et al. [108] report nutraceutical properties for *Apium nodiflorum*, and propose it as a potential new crop, an interesting activity against intestinal parasites has been reported for *Origanum virens* [109], used as a condiment, and a position as a commercial spice is proposed for *Crithmum maritimum*, after describing a chemical composition compatible with nutraceutical properties [110]. These medicinal properties provide WFP with an added value for their local consumption that may be used in the development of new, healthy food.

Studies about the nutrients and the bioactive compounds of the WFP are very important to demonstrate the big potential of these plants as nutraceuticals. Sánchez-Mata et al. [17] have carried out several nutritional studies on WFP with very interesting results, some of them related with the plants quoted in this meta-analysis. Examples of that are *Rumex pulcher* L. with a level of vitamin B_9_ (folic acid) higher than the 100% daily recommended intakes; *Urtica dioica*, *Arbutus unedo*, *Chenopodium album* L., *Capsella bursa-pastoris* (L.) Medik. and *Eruca vesicaria* (L.) Cav. with levels of vitamin C higher than the 100% recommended intakes; *Celtis australis* L., *Parietaria officinalis* L. subsp. *judaica* (L.) Béguinot and *Urtica dioica* with calcium sources higher than the 50% daily advised; or *Celtis australis*, *Sanguisorba minor* Scop. and *Chenopodium album* also with values of magnesium higher than those recommended.

### 3.7. Particularly Representative Plants and Plants Associated with Believes

Some WFP, basically, but not only, among the most quoted ones, may be considered iconic in terms of their particularly relevant use on a quotidian basis and for the appreciation people has of them. Three clear examples are *Laurus nobilis*, *Origanum vulgare* and *Thymus vulgaris*, with their abundant daily use as condiment (among other not so frequent uses), equalling or overcoming the use of very important condiments from foreign, cultivated plants, such as *Capsicum annuum* L. and *Piper nigrum* L. Similarly, but to a slightly lesser extent, another aromatic plant, *Rosmarinus officinalis*, has also a place among the most habitual used plants, also as a condiment, completing, with *Thymus* and *Laurus*, a classical *bouquet garni*. These plants, in their daily use, are not the protagonists of the dishes in terms of volume or weight, but many popular, typical -from old or modern tradition- and highly appreciated dishes (soups, rice, stews, roasts, salads, even pizzas) could not be conceived without them. Moving to sweet, we find a similar example. To give the necessary and distinctive flavour to ‘*bunyol*’ or ‘*brunyol’* (fritter), very typical of Easter week and nowadays eaten all along the year, if cultivated *Pimpinella anisum* L. fruits are not available, or if others are preferred to confer the anis smell and taste, *Carum carvi* L. or *Foeniculum vulgare* subsp. *piperitum* fruits are used.

Other plants may also be considered as very appreciated, but are not consumed daily, mainly due to their seasonality. This is the case of *Rubus ulmifolius* fruits, eaten directly or prepared in jam, and, most relevantly, of *Asparagus acutifolius* spears, collected in big amounts every spring and eaten boiled or in salad, soup or, mostly, omelette.

Some WFP are associated with magic or religious believes, and, to some extent, fall within the concept of taboo, which is frequently related to foods [111]. All *Ruta* species are claimed to be abortive, and this is usually maintained as an old secret, but they are consumed as foods, although with the warning of this possible side effect. A very common tradition in the area studies consists in blessing leafed branches of the above-mentioned *Laurus nobilis* (and, not so commonly *Thymus vulgaris* and *Rosmarinus officinalis*) in the Palm Sunday celebration, and the belief that these blessed branches protect the house and its inhabitants is general. It is not at all rare, in addition, to consider that blessed *Laurus* is better than normal one for cooking, expressed with sentences such as “*millor si està beneït el dia de Rams*” (‘better if blessed during Palm Sunday’) [100]. In this case it is not difficult to deduce that, irrespective of the believes, this was a manner of replacing the existences of last year’s *Laurus* leaves for condiment with the new ones, richer in essential oils. In the same magic or religious field, some wild *Allium* species, such as *A. ampeloprasum* L., are called ‘*all de bruixa*’ (‘witch’s garlic’) and are not consumed by some people, who associate the plant to witches and toxicity. Associated with another kind of believes, some people has the opinion that consuming nowadays plants collected in the particularly in a direct manner in salads, is dangerous because they are reached by pesticides and other chemical products.

### 3.8. Uses Beyond the Home: From Past to Future, with Some Case Examples

Recovering or expanding the use of old foods, in many cases applying the so-called slow food or zero-kilometre cuisine paradigms, is often associated with the concept of ‘*terroir*’, closely linked to traditional knowledge on the food products (importantly of plant or fungal origin) [112,113]. This tendency has been termed as ‘nostalgic’ (‘nostalgia of ‘*terroir*’, [114]; ‘nostalgic foods’, [115]), not so much in a pejorative sense, but because of the idea of returning to remembered and appreciated sources, with the added value of generating identity. In an anthropological study of Mallorca’s cuisine (one of the territories concerned in the present paper), Trias [116] described a desire for food fully imbued with cultural meaning, beyond simply covering the primary nutritional needs. The identity can be created with cultivated plants of foreign origin, as is the case of one of the leaders of Catalan cuisine, ‘*pa amb tomata*’, called in some places ‘*pa amb oli*’ (‘bread with tomato’, ‘bread with oil’, prepared by rubbing the bread with tomato and seasoning it with olive oil) [117]. Apart from that, WFP may also play this role of generating identity. For instance, *Molopospermum peloponnesiacum* (Figure 6), appreciated to quite an extent in all Catalan eastern Pyrenees, is considered ‘the Catalans’ salad’ in Northern Catalonia or French Eastern Pyrenees department [118].

These nostalgic or identity issues make WFP recoverable. In fact, regarding WFP for salad, some of them were considered deprivation-time food, but this statement can be nuanced and complemented: for instance, talking about *Reichardia picroides*, we recorded this information: “*Abans hi havia necessitat, però també agradaven. Hi ha avis que encara les persegueixen*” (‘earlier, there was a need, but they were also liked. There are some elderly people that still look for them’) [97]. It was not only famine, but taste as well. Indeed, the 85-year-old brother of one of our informants (Supplementary Material S2), regularly ate *Portulaca oleracea* salad every summer at his uncles’ house, until he was 12–13 years old. Since then he has never eaten it again. He professed that he did not miss the salad, but he would not mind trying it again, because it was tasty. The popularity of this plant and, at the same time, the lack if its current use, is confirmed by the 67-year-old son of one of our informants (Supplementary Material S2) who stated that, when he was 6, 7 or 8 years old, one man used to go regularly to his father’s orchard to harvest *Portulaca oleracea* for familiar consumption, but that he did not eat it since a lot of years. Similarly, the use of some plants has definitely or importantly declined. This is the case of eating *Rumex crispus* L., even directly in the field, or the consumption of *Carlina acanthifolia* All. subsp. *cynara* (Pourr. ex Duby) Arcang. as bread. Also the use has at least almost disappeared of *Althaea officinalis* L. root to give consistency to a broth, replaced by modern culinary densifiers. Conversely, some used arise as new in the area in recent times, such the one of *Papaver* seeds in bread and similar products. Other uses come from old times and are maintained. For instance, collecting some WFP such as *Asparagus acutifolius*, *Crithmum maritimum* and *Rubus ulmifolius* for food purposes continues to be very much alive, because people like to eat them (see also Section 3.7). Similarly, sucking *Foeniculum vulgare* inflorescence directly in the field continues alive. For *Crithmum maritimum*, a very common and abundant plant, a protection status has even been established by the government of the Balearic Islands, with personal-use collection allowed, and a permission needed when collecting for commercial purposes [119]. The different reasons stated above (cultural and identity relevance, not-forgotten good taste, economic value), united to sustainable or zero kilometre or slow food currents, give the opportunity of placing at least some WFP onto the market. We will expound, in a qualitative manner, a few case examples of the area studied.

#### 3.8.1. Markets and Industrialised Products

**Figure 7:**
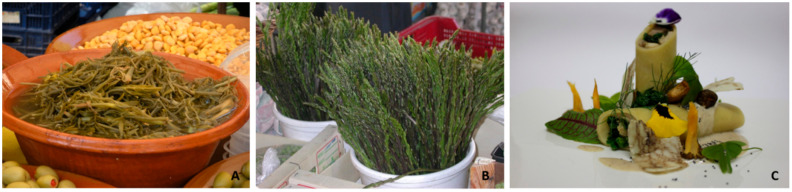
Examples of wild food plants present in markets (**A**,**B**) or restaurants (**C**). (**A**) *Crithmum maritimum*; (**B**) *Asparagus acutifolius*; (**C**) *Urtica dioica*.

Some WFP are regularly sold in local food markets, which is a first step towards commercialisation, with the added value of being seasonal products. These local markets are not always close to the places where plants have been collected. *Taraxacum dissectum*, a Pyrenean endemic, is sold in Barcelona, particularly in one of its most important markets, visited by locals, but also by numerous international people: “*es venen al mercat, sobretot a la Boqueria, en parades especialitzades. Venen del Pirineu*” (‘they are sold in the market, particularly in la Boqueria, in specialised stalls. They come from the Pyrenees’) [95]. Some other species, such as *Asparagus acutifolius* and *Thymus vulgaris*, are commonly sold in markets. *Portulaca oleracea*, very common as food -and cultivated- in France, has not been a big success currently is Spain, but we have observed it in one market in NE Catalonia [120] (Figure 7), where the seller told us that this was a weed that used to be eaten and, since he had to harvest it to take care of the orchard, he preferred to try to sell it. The food composition data available for this species [17] and the research suggesting its quality or nutraceutical with, among others, antioxidant activity [35,36,37,121], together with the information on past and possible future consumption of the plant in the present paper, make it a good candidate for new food development in the territories considered and neighbouring areas (except for people with genitourinary troubles, due to its high content of oxalates). Among others, WFP such as *Helianthus tuberosus* L., *Fragaria vesca* and *Rubus idaeus* are regularly present in markets in the area studied, as well.

Not all plants are presented just fresh in the markets. *Crithmum maritimum*, very common all along the coast in the whole territory studied, is practically only consumed on Mallorca, and is sold preserved in water, wine vinegar and salt (Figure 7). This preserve has advanced to the industrial phase, and several firms prepare, pack and commercialise it. A similar process to this industrialisation is the cultivation, in Catalonia, of *Cynara cardunculus*, established with an agreement to sell to companies of the dairy sector to elaborate curd, cheese and similar products. Semi-industrial jams of *Arbutus unedo* or *Rosa canina* fruits are often sold in traditional markets. Several projects dealing with WFP and implying the commercialisation of their products are available at https://www.eixarcolant.cat/directori.

#### 3.8.2. Restaurants

Another indicator of WFP expansion beyond the local tradition is their transference from home kitchens to restaurants, in some cases, but not always, located near the place where one or another WFP is collected and popularly consumed. Some WFP of the studied area regularly reach the restaurants’ menu, as in the markets usually as seasonal dishes, but on some occasions all year round. The latter is the case of condiments, such as *Laurus nobilis*, *Origanum vulgare*, *O. virens*, *Rosmarinus officinalis*, *Satureja montana*, *Thymus piperella* or *T. vulgaris* leaves (Supplementary Material S2), kept dry throughout the year and, in some cases, with WFP cultivated on a small scale near the restaurant. In any case, the former are more interesting, in the sense that they play a protagonist role in some dishes. Omelettes, or other preparations of *Asparagus acutifolius,* are frequent in spring (Supplementary Material S2). A reasonable number of restaurants have contacts with people who collect the plants exclusively for them. Salads based on *Molopospermum peloponnesiacum* or *Taraxacum dissectum* also appear in some menus in spring.

Between spring and summer, we have been told that *Sambucus nigra* inflorescence’s fritters may be at least occasionally found. In the same period of time, *Thymus vulgaris* flowers are used to prepare a sorbet as a dessert (Supplementary Material S2), and in similar forms, the fruit of *Opuntia maxima* Mill.

In littoral areas, *Salicornia patula* is used in fish dishes. On the island of Mallorca preserved leaves of *Crithmum maritimum* are frequently offered, during the whole year, as an aperitif or so-called *tapa*, and flowers and stems of *Allium triquetrum* L. appear more scarcely in salads. In areas of Catalonia, *Allium roseum* L., *Chondrilla juncea* L. and *Rumex crispus* are also served in restaurants. Other plants such as *Beta vulgaris* subsp. *maritima* (L.) Arcangeli, *Chenopodium album*, *Diplotaxis erucoides* (L.) DC., *Lactuca serriola* L., *Malva sylvestris*, *Papaver rhoeas*, *Plantago lanceolata* L., *Rumex pulcher*, *Silene vulgaris* and *Urtica dioica* have regularly been furnished to restaurants (Supplementary Material S2).

There are more case examples, and there could in fact be many more. We here explain just one that we have never seen in a market nor in a restaurant, but that we are convinced could be highly appreciated: *Chenopodium bonus-henricus*. During eight years (2004–2011), the senior author of this paper was the co-responsible person for a subject for undergraduate pharmacy and biology students on useful plants of the Catalan Pyrenees, mostly based on field work. Every year, within the framework of this subject, he cooked an omelette with the leaves of this plant, which he proposed the students to taste. In all cases not one of these young people had eaten this plant, but unanimously expressed their appreciation of the omelette, for many of them better than the classical one of another plant from the same Amaranthaceae family, *Spinacia oleracea*. In the Italian Alps, the species is used in a similar way to its use in the Catalan linguistic area [122]. Several *Chenopodium* species, but not this one, appear as interesting Mediterranean WFP [17]. Research on the food composition of this species would surely result in possibilities of its utilisation on a larger scale than nowadays.

The above-mentioned examples regarding the reach of WFP in markets and restaurants are only some without any claim of completeness, which account for the vitality of the traditional knowledge and use of wild plants as food. The presence in restaurants makes it possible the taste of WFP can be experienced by people interested in gastronomy and unfamiliar cuisine, and means in many cases that touristic resorts are furnished with WFP. This opens to the tourists (probably starting for those using rural tourism facilities) the access to WFP, enlarging their reach beyond local people.

Science starts being considered in gastronomy, which, in its turn, is starting to be considered a science (see a reference to a Science & Cooking congress in the concluding remarks). In this context, traditional knowledge on WFP, recorded by a discipline at the interface of social and natural sciences, has a very high value. If, as stated in the introduction, the *nouvelle cuisine* started with an ethnobotanical basis, now culinary facilities are interested on WFP and in combining tradition and innovation. Additionally, some food products, such as the whole Mediterranean diet—to quote the one concerning the area studied in this paper—are inscribed on the UNESCO’s list of intangible cultural heritage. This is why restaurants, including high-gastronomy ones, often have contacts with people (not rarely botanists) who provide them with WFP. Further research on these aspects would most probably reveal many more cases than those we have quoted here of traditional WFP present in restaurants or touristic resorts, or permit the introduction, in the fields mentioned, of other WFP, thereby enlarging their utility.

## 4. Concluding Remarks

The results and data analyses performed in the present paper show an important pool of 278 WFP traditionally used in the Catalan linguistic area. The dataset shows a significant consistency and robustness as assessed through several quantitative ethnobotany indexes. Further research should be performed, on the one hand to fill some territorial gaps in order to cover all the territory, and on the other hand to incorporate into the database information from historical and anthropological research (such as the culinary corpuses of Catalonia [123,124] or of Valencia [125], rich on WFP information), which would enrich the already important pool of data on WFP in the Catalan-speaking territories.

In the area considered, WFP are not only remnants of the past, but a treasure trove from the past, still alive today, constituting a very valuable resource for the future. We believe that the examples we have provided clearly show that WFP ethnobotany is one of the most appropriate approaches and bases for selecting candidates for new food development. Adding to this the information from Talavera’s (unpubl. res.) surveys on people’s appreciation of WFP, it is quite likely that some taxa will emerge as candidates to be counted among the foods of the future, based on current research focused on the past traditional knowledge of plants. Throughout the present paper, we have underlined some taxa (*Apium nodiflorum*, *Crithmum maritimum*, *Reichardia picroides*) that had already been proposed to be launched as new crops or new commercial plants on a scale larger than that of local use. Our results fully reinforce and support these proposals. Some other WFP species could be added to the proposal list, among others the few we will briefly comment now. *Helianthus tuberosus* and *Portulaca oleracea*, already profusely cultivated and sold in France, could enlarge their market area. *Salicornia patula* could be introduced in markets and restaurants, probably collected from the wild, as in the case of *Crithmum maritimum* and similarly to what we have observed to happen in France with this and related taxa. *Origanum virens* could increase its market and could be cultivated. For this and other aromatic and condiment plants, successful cultivation tests have been performed [126]. *Chenopodium bonus-henricus* and *Urtica dioica* could constitute, either collected from the wild or cultivated, interesting commercial products. Public administrations’ help would be suitable for initiating the development of the crops and products mentioned. Apart from the economic interest for people involved, these new plants and products put on the market will contribute, in general, to food security and, particularly given their local-origin component and traditional knowledge basis, to food sovereignty.

During the Science & Cooking World Congress, held in Barcelona in 2019, the manifesto “Scientific gastronomy Barcelona 2019” [127] vindicated this concept, understood as culinary and gastronomic science. From the present paper and analogous ones, it is undoubted that ethnobotany, and particularly the research on WFP, may relevantly contribute to this innovative plot of knowledge.

## Figures and Tables

**Table 1:** The top 30 taxa and their preparation forms.

Taxon, Family and Herbarium Voucher	Catalan Names	Used Part	Preparation Form	Total UR	CI	PFAF Validation	FFF Validation
*Thymus vulgaris* L. (Lamiaceae) BCN 96,764	Farigola, tem, timó, timó femella, timó mascle, timonet, timonet femella, timonet mascle, tremoncell	Cortical parenchyma, flower, flowering stem, flowering top, leaf, inflorescence, aerial part, flowering aerial part, stem with leaves/branches, young aerial part	Air dried, boiled in water, boiled in water and oil, condiment, cooked in oil, preserved in brine, raw, unknown	957	0.52	*	*
*Foeniculum vulgare* Mill. subsp. *piperitum* (Ucria) Cout. (Apiaceae) BCN 125,404	Fenoll, fonoll	Aerial part, bulb, fruit, flower, flowering aerial part, inflorescence, infructescence, leaf, ripe fruit, root, seed, stem, stem with leaves/branches, unknown, young aerial part, young leaf	Air dried, boiled in water, boiled in water and fat, boiled in water and oil, condiment, cooked in oil, preserved in brine, preserved in oil, raw, unknown	482	0.24	*	*
*Laurus nobilis* L. (Lauraceae) BCN 150,355	Llaurer, llor, llorer, llort	Fruit without seeds, leaf	Air dried, boiled in water and oil, condiment, preserved in oil, preserved in vinegar, raw, unknown	475	0.27	*	
*Rubus ulmifolius* Schott (Rosaceae) BCN 156,557	Albarzer, abatzer, barder, barsa, barser, esbarzer, romeguera, romiguera, verder	Fruit, infructescence, ripe fruit, stem, young aerial part, young shoot	Boiled in water, condiment, cooked in sugar, raw, unknown	425	0.25	*	
*Mentha spicata* L. (Lamiaceae) BCN 125,414	Herba de Santa Maria, herba-sana, herba-sana vera, menta, menta de bou, menta de les faves, menta del consol, terongina	Aerial part, flowering top, leaf, unknown, young leaf	Air dried, boiled in water, condiment, raw, unknown	415	0.23	*	*
*Rosmarinus officinalis* L. (Lamiaceae) BCN 156,603	Romaní, romanyí, romer, romer femella, romer mascle	Aerial part, flower, flowering aerial part, flowering stem, flowering top, inflorescence, leaf, stem with leaves/branches, unknown	Boiled in water, condiment, raw, unknown	356	0.21	*	*
*Origanum vulgare* L. (Lamiaceae) BCN 125,392	Herbamusca, moraduix, moraduix bord, moraduix de la Mare de Déu, orenga, roca morera	Aerial part, flowering aerial part, flowering top, inflorescence, leaf, unknown	Condiment, raw, unknown	341	0.20	*	*
*Ficus carica* L. (Moraceae) BCN 150,361	Figuer, figuera	Fruit, infructescence, latex, leaf, ripe fruit, stem, unknown, young aerial part, young shoot	Air dried, boiled in milk, boiled in water, condiment, cooked in sugar, curd, raw, toasted, unknown	317	0.17	*	
*Satureja montana* L. (Lamiaceae) BCN 125,403	Herba de les olives, saboritja, sajolida, salseta de pastor	Aerial part, flowering aerial part, flowering top, inflorescence, leaf, cortical parenchyma, stem with leaves/branches, unknown	Condiment, preserved in brine, unknown	285	0.14	*	*
*Reichardia picroides* (L.) Roth (Asteraceae) BCN 113,704	Cosconia, cosconilla, llicsó	Aerial part, leaf, unknown, young aerial part, young leaf	Boiled in water, condiment, cooked in oil, raw, unknown	249	0.15	*	
*Arbutus unedo* L. (Ericaceae) BCN 96,768	Alborcer, arboç, arbocer, cierre d’arboç, cirerer de pastor, llipoter	Fruit, fruit without seeds, ripe fruit, unknown	Boiled in water, condiment cooked in sugar, preserved in high-grade alcohol, raw, unknown	234	0.14	*	
*Cynara cardunculus* L. (Asteraceae) BCN 29,860	Card, card coler, card de fer formatge, card de formatjar, card formatger, carxofera borda, carxofera de presó, escard de formatjar, herba presonera, herbacol, preor, presor, presora	Aerial part, flower, leaf, leaf stalk, stem, stem with leaves/branches, unknown	Boiled in water, condiment, curd, preserved in brine, raw, unknown	222	0.13	*	
*Chondrilla juncea* L. (Asteraceae) BCN 150,377	Cama-roja, enciam, escanyaguineus, masteguera, màstec, xicoia	Leaf, young aerial part, unknown, whole plant, young leaf	Boiled in water, condiment, cooked in oil, preserved in vinegar, raw, unknown	220	0.13	*	
*Silene vulgaris* (Moench) Garcke (Caryophyllaceae) BCN 96,770	Colís, colitx, coniell, conillet, esclafidor, petador, petapetons, verdura	Aerial part, flower, flowering top, leaf, root, stem, unknown, young aerial part, young leaf	Boiled in water, boiled in water and fat, boiled in water and oil, condiment, cooked in oil, preserved in vinegar, raw, toasted, unknown	198	0.10	*	
*Portulaca oleracea* L. (Portulacaceae) BCN 46,835	Enciam de frare, enciam de patena, verdolaga	Aerial part, leaf, flower, unknown, young aerial part, young leaf, young shoot	Boiled in water, boiled in water and oil, condiment, cooked in oil, preserved in vinegar, raw, toasted, unknown	191	0.12	*	
*Urtica dioica* L. (Urticaceae) BCN 29,814	Estrígol gran, ortiga, ortiga barragana, ortiga blanca, ortiga de bou, ortiga de llei, ortiga de mare, ortiga de riu, ortiga gran, ortiga grossa, ortiga major, ortiga negra	Aerial part, flowering top, leaf, unknown, young aerial part, young leaf	Boiled in water, boiled in water and oil, condiment, cooked in oil, raw, unknown	188	0.11	*	*
*Taraxacum officinale* Weber in Wiggers (Asteraceae) BCN 150,371	Angelets, apagallums, dent de lleó, herba amarga, lletsó, lletsó d’ase, queixal de verro	Aerial part, inflorescence, leaf, root, unknown, whole plant, young aerial part, young leaf	Boiled in water, condiment, cooked in oil, cooked in sugar, raw, unknown	173	0.10	*	*
*Asparagus acutifolius* L. (Asparagaceae) BCN 125,479	Espargolera, esparreguera, esparreguera borda, esparreguera silvestre	Stem, unknown, young shoot	Boiled in water, boiled in water and oil, cooked in oil, raw, unknown	145	0.09	*	*
*Cichorium intybus* L. (Asteraceae) BCN 296,600	Cama-roja, cosconilla, endívia, escarola borda, fuell, màstec, màstec bord, masteguera, raditxa, xicoia, xicoira, xicòria	Aerial part, inflorescence, leaf, root, seed, unknown, young aerial part, young leaf	Boiled in water, boiled in water and oil, cooked in oil, raw, toasted, unknown	145	0.08	*	
*Sorbus domestica* L. (Rosaceae) BCN 150,384	Moixera, server, servera, serverola	Fruit, ripe fruit	Air dried, boiled in water and oil, cooked in sugar, raw, unknown	143	0.09	*	
*Papaver rhoeas* L. (Papaveraceae) BCN 24,940	Babol, gallaret, gallgallaret, paparota, pipiripip, quiquiriquic, rosella	Aerial part, leaf, seed, unknown, young aerial part, young leaf	Boiled in water, condiment, cooked in oil, raw, unknown	140	0.09		*
*Thymus serpyllum* L. (Lamiaceae) BCN 25,019	Brotònica, essència de pastor, farigola, farigola borda, farigola de muntanya, farigola de pastor, folcó, salseta de pastor, serpoll, timó, timonet bord	Aerial part, flowering aerial part, flowering top, inflorescence, nectar, unknown	Boiled in water, condiment, raw, unknown	121	0.07		*
*Rorippa nasturtium-aquaticum* (L.) Hayek *nasturtium-aquaticum* (Brassicaceae) BCN 24,971	Api bord, réixec, créixen, gréixol	Aerial part, leaf, stem, unknown, whole part, young aerial part, young leaf	Boiled in water, cooked in oil, preserved in vinegar, raw, unknown	118	0.07		*
*Molopospermum peloponnesiacum* (L.) Koch (Apiaceae) BCN 24,934	Api bord, coscó, coscoll	Aerial part, fruit, leaf, stem, unknown, young leaf, young shoot	Cooked in sugar, raw, unkwnown	111	0.07		
*Borago officinalis* L. (Boraginaceae) BCN 68,582	Borraina, borratja, borratxa, pa amb vi	Aerial part, flower, leaf, stem	Boiled in water, boiled in wáter and oil, cooked in oil, raw, unkwnon	109	0.05	*	*
*Rubus ideaeus* L. (Rosaceae) BCN 24,977	Gerdera, gerdoner, gerdonera, gerguer, gerguera, jordonera	Fruit, infructescence, ripe fruit, unknown	Condiment, cooked in sugar, raw, unknown	109	0.07	*	*
*Celtis australis* L. (Cannabaceae) BCN 24,741	Lledoner	Fruit, ripe fruit	Raw, unknown	108	0.07		*
*Sambucus nigra* L. (Adoxaceae) BCN 24,984	Benabre, bonabre, bonarbre, menabre, sabuc, sabuquer, saüc, sauguer, saüger, saüquer, sauquer, suc, suguer, suquer	Flowering aerial part, fruit, inflorescence, ripe fruit, seed	Boiled in water, condiment, cooked in oil, cooked in sugar, cooked with wine, raw, unknown	103	0.06		*
*Sonchus oleraceus* L. (Asteraceae)BCN 25,008	Llecsó, llenteín, llepsó, lletsó, llicsó, llitsó	Aerial part, leaf, unknown, young aerial part, young leaf	Raw, unknown	102	0.06		*
*Fragaria vesca* L. (Rosaceae) BCN 24,889	Maduixa de bosc, maduixa silvestre	Fruit, immature fruit, infructescence, ripe fruit	Cooked in sugar, preserved in wine, raw, unknown	94	0.06		*
Mean ± SD (30 top taxa)				242.53 ± 179.11	0.14 ± 0.09		
Mean ± SD (all taxa)				35.58 ± 94.02	0.02 ± 0.05		

BCN: Herbarium of the Centre de Documentació de Biodiversitat Vegetal,* Universitat de Barcelona*. UR: Use reports. CI: Cultural importance index [70]. In the two last columns, the asterisk (*) indicates validation by the sources mentioned. FFF: folk functional food ([39]; validation: [105]). PFAF: Plants for a Future database (validation: www.pfaf.org).

**Table 2:** The 10 main kinds of traditional foods and examples of wild food plants involved in each case.

Kind of Traditional Food	Synthetic Explanation	Examples of Plants Involved
Cake	Basic preparation of the cake with flour, butter or olive oil and water (and sugar or salt depending on the kind of cake), and addition of the plant inside or in the surface	*Fragaria vesca*, *Papaver rhoeas*, *Silene vulgaris*
Condiment	Used to season a salad, a fritter, a pizza or a meat or fish roast or stew	*Carum carvi*, *Laurus nobilis*, *Origanum vulgare*, *Thymus vulgaris*
Fritter	Put in batter and fried in olive oil, with salt or sugar depending on the use as meat dishes’ complement or as a dessert	*Sambucus nigra*
Omelette	Slightly fried in olive oil or sauté, mixed with eggs, and fried in olive oil	*Asparagus acutifolius*, *Chenopodium bonus-henricus*, *Malva sylvestris*, *Urtica dioica*
Preserve	Cooked in sugar, preserved in brine or preserved in vinegar	*Arbutus unedo*, *Crithmum maritimum*, *Foeniculum vulgare*, *Rubus ulmifolius*
Salad	Cleaned and seasoned with olive oil and sometimes salt and/or condiments	*Molopospermum peloponnesiacum*, *Portulaca oleracea*, *Reichardia picroides*, *Taraxacum dissectum*
Soup	Boiled with water, in some cases with bread and an egg added at the end of the preparation	*Mentha spicata*, *Thymus vulgaris*, *Urtica dioica*
Stew	Stewed with meat	*Castanea sativa*
Sweet delicacy	Cleaned and directly consumed, often in the field just after collection	*Arbutus unedo*, *Celtis australis*, *Prunus spinosa*, *Rubus idaeus*, *Rubus ulmifolius*, *Fragaria vesca*
Vegetable	Boiled in water	*Malva sylvestris*, *Silene vulgaris*, *Urtica dioica*

## Data Availability

The data presented in this study are available on request from the corresponding authors. The data are not yet publicly available because authors database starts its public access in February 2021 with medicinal plant uses’ information. Information on food plants is foreseen to be publicly available on February 2022.

## Supplementary Materials

The following are available online at https://www.mdpi.com/2304-8158/10/1/61/s1; Supplementary Material S1. Studied areas and research works analysed in the present paper. Supplementary Material S2: Information not coming from the database analysed, but from complementary informants. Supplementary Material S3: Wild food plants and their uses in the Catalan linguistic area (simplified data). Supplementary Material S4: Wild food plants and their uses in the Catalan linguistic area (complete database).
