# Supplementary material for: The Power of Wild Plants in Feeding Humanity: A Meta-Analytic Ethnobotanical Approach in the Catalan Linguistic Area
Source: Foods. 2020 Dec 29;10(1):61. doi: 10.3390/foods10010061 (PMC7824323; doi:10.3390/foods10010061)
Supplement: Supplementary file 1 [file foods-10-00061-s001.zip › Supplementary material 2.docx]

**Supplementary material 2.** Information in subheading 3 of the present work coming from the nine brief, directed interviews performed in September 2020, including the names of the informants in each case. The information pieces are ordered according to their appearance in the text

| **Information** | **Informants** |
| --- | --- |
| *Origanum virens* and *Thymus piperella* as representative of a particular geographical zone (southern Valencian area) where, in addition, very often they incorporate elements of local identity | Albert Sala |
| *Portulaca oleracea* as a plant quite appreciated in old times, which could be tasted again nowadays without problems | Esteve Roura, Esteve Clavaguera |
| *Laurus nobilis*, *Origanum vulgare*, *O. virens*, *Rosmarinus officinalis*, *Satureja montana*, *Thymus piperella* and *T. vulgaris* as plants currently being sold in markets or present in restaurants’ menus in a regular manner (used as condiments) | Ester Bronsoms, Joan Manté, Catalina Planas, Albert Sala, Jordi Subirós, |
| Omelettes or other preparations of *Asparagus acutifolius* as present in restaurants in spring | Joan Manté, Jordi Subirós |
| *Thymus vulgaris* flowers as used to prepare a sorbet as a dessert in restaurants in spring and summer | Joan Manté, Jordi Subirós |
| Plants such as *Beta vulgaris* subsp. *maritima*, *Chenopodium album*, *Diplotaxis erucoides*, *Lactuca serriola*, *Malva sylvestris*, *Papaver rhoeas*, *Plantago lanceolata*, *Rumex pulcher*, *Silene vulgaris* and *Urtica dioica* as regularly furnished to restaurants | Guillem Figueras, Natacha Filippi |
