# Supplementary material for: The Power of Wild Plants in Feeding Humanity: A Meta-Analytic Ethnobotanical Approach in the Catalan Linguistic Area
Source: Foods. 2020 Dec 29;10(1):61. doi: 10.3390/foods10010061 (PMC7824323; doi:10.3390/foods10010061)
Supplement: Supplementary file 1 [file foods-10-00061-s001.zip › Supplementary material 3.docx]

**Supplementary material 2.** Wild food plants and their uses in the Catalan linguistic area (simplified data).

| **Taxon** | **Used part** | **Mode of preparation** |
| --- | --- | --- |
| *Abies alba* Mill. | Cone | Unknown |
| *Achillea millefolium* L. | Flowering top | Preserved in high-grade alcohol |
| *Allium ampeloprasum* L. | Aerial part | Boiled in water |
|  |  | Boiled in water and oil |
|  | Stem | Boiled in water |
|  |  | Unknown |
|  | Young aerial part | Boiled in water |
| *Allium paniculatum* L. | Aerial part | Boiled in water and oil |
| *Allium roseum* L. | Aerial part | Boiled in water and oil |
|  |  | Cooked in oil |
|  |  | Raw |
|  | Bulb | Raw |
|  | Unknown | Condiment |
| *Allium schoenoprasum* L. | Bulb | Cooked in oil |
|  | Stem | Condiment |
| *Allium sphaerocephalon* L. | Aerial part | Unknown |
| *Allium triquetrum* L. | Whole plant | Raw |
| *Allium vineale* L. | Aerial part | Boiled in water and oil |
| *Althaea officinalis* L. | Root | Condiment |
| *Amaranthus* sp. | Seed | Unknown |
| *Amelanchier ovalis* Medic. | Fruit | Raw |
|  | Ripe fruit | Raw |
|  | Unknown | Unknown |
| *Ampelodesmos mauritanica* (Poiret) T.Durand et Schinz | Stem | Raw |
| *Anemone hepatica* L. | Leaf | Unknown |
| *Anethum graveolens* L. | Aerial part | Condiment |
|  | Fruit | Condiment |
|  | Seed | Condiment |
|  | Young leaf | Condiment |
| *Angelica sylvestris* L. | Leaf | Raw |
| *Anthemis arvensis* L. | Leaf | Boiled in water |
| *Anthriscus cerefolium* (L.) Hoffm. | Leaf | Condiment |
| *Antirrhinum majus* L. subsp. *majus* | Inflorescence | Raw |
| *Aphyllanthes monspeliensis* L. | Aerial part | Raw |
|  | Flower | Raw |
|  | Inflorescence | Raw |
|  | Unknown | Unknown |
| *Apium nodiflorum* (L.) Lag. subsp. *nodiflorum* | Aerial part | Boiled in water |
|  |  | Raw |
|  | Young aerial part | Raw |
| *Arbutus unedo* L. | Fruit | Condiment |
|  |  | Cooked in sugar |
|  |  | Preserved in high-grade alcohol |
|  |  | Raw |
|  |  | Unknown |
|  | Fruit without seed | Boiled in water |
|  | Ripe fruit | Cooked in sugar |
|  |  | Raw |
|  | Unknown | Boiled in water |
|  |  | Raw |
| *Arctium minus* Bernh. | Leaf | Unknown |
|  | Stem | Boiled in water |
| *Arctostaphylos uva-ursi* (L.) Spreng. | Fruit | Unknown |
| *Artemisia abrotanum* L. | Leaf | Condiment |
| *Artemisia absinthium* L. | Flowering top | Condiment |
|  | Leaf | Condiment |
|  |  | Preserved in high-grade alcohol |
| *Artemisia alba* Turra | Aerial part | Condiment |
| *Artemisia arborescens* L. | Leaf | Condiment |
| *Artemisia campestris* L. subsp. *glutinosa* (Gay ex Bess.) Batt. in Batt. et Trab. | Aerial part | Condiment |
| *Artemisia chamaemelifolia* Vill. | Flowering top | Condiment |
| *Artemisia verlotiorum* Lamotte | Unknown | Raw |
| *Arum italicum* Mill. italicum | Leaf | Boiled in water |
| *Arundo donax* L. | Leaf | Condiment |
|  | Young shoot | Condiment |
| *Asparagus acutifolius* L. | Stem | Boiled in water |
|  |  | Cooked in oil |
|  |  | Unknown |
|  | Unknown | Cooked in oil |
|  | Young shoot | Boiled in water |
|  |  | Boiled in water and oil |
|  |  | Cooked in oil |
|  |  | Raw |
|  |  | Unknown |
| *Asparagus albus* L. | Young shoot | Unknown |
| *Asparagus horridus* L. in J.A.Murray | Young shoot | Boiled in water |
|  |  | Boiled in water and oil |
|  |  | Cooked in oil |
|  |  | Raw |
|  |  | Toasted |
|  |  | Unknown |
| *Asparagus officinalis* L. | Stem | Toasted |
|  | Young shoot | Boiled in water |
|  |  | Boiled in water and oil |
|  |  | Cooked in oil |
|  |  | Raw |
|  |  | Unknown |
| *Asphodelus fistulosus* L. | Bulb | Unknown |
|  | Leaf | Cooked in oil |
| *Asplenium septentrionale* (L.) Hoffm. | Frond | Raw |
| *Astragalus baeticus* L. | Fruit | Toasted |
|  | Seed | Unknown |
| *Atractylis cancellata* L. | Floral bud | Unknown |
|  | Inflorescence | Boiled in water and oil |
| *Bellis perennis* L. | Young leaf | Raw |
| *Beta vulgaris* L. subsp. *maritima* (L.) Arcang. | Leaf | Boiled in water |
|  |  | Cooked in oil |
|  |  | Unknown |
|  | Unknown | Unknown |
|  | Young leaf | Raw |
| *Betula pendula* Roth | Sap | Unknown |
| *Borago officinalis* L. | Aerial part | Boiled in water |
|  |  | Boiled in water and oil |
|  |  | Unknown |
|  | Flower | Cooked in oil |
|  |  | Raw |
|  |  | Unknown |
|  | Leaf | Boiled in water |
|  |  | Boiled in water and oil |
|  |  | Cooked in oil |
|  |  | Raw |
|  |  | Unknown |
|  | Leaf stalk | Raw |
|  | Stem | Condiment |
|  |  | Raw |
|  |  | Unknown |
|  | Stem with leaves / branches | Boiled in water |
|  |  | Cooked in oil |
|  | Unknown | Boiled in water |
|  |  | Cooked in oil |
|  |  | Raw |
|  |  | Unknown |
|  | Young aerial part | Raw |
| *Brachypodium retusum* (Pers.) Beauv. | Young aerial part | Cooked in oil |
| *Brassica juncea* (L.) Czern. | Leaf | Raw |
| *Bryonia cretica* L. subsp. *dioica* (Jacq.) Tutin | Unknown | Cooked in oil |
|  |  | Unknown |
|  | Young aerial part | Cooked in oil |
|  | Young shoot | Condiment |
|  |  | Cooked in oil |
| *Calendula arvensis* L. | Inflorescence | Raw |
|  | Leaf | Raw |
|  | Unknown | Raw |
| *Calendula officinalis* L. | Floral bud | Unknown |
|  | Flower | Condiment |
|  |  | Raw |
|  | Inflorescence | Raw |
|  | Leaf | Raw |
|  | Young aerial part | Boiled in water and oil |
|  |  | Raw |
| *Calluna vulgaris* (L.) Hull | Flower | Raw |
| *Campanula rapunculus* L. | Flower | Raw |
|  | Leaf | Raw |
|  |  | Unknown |
|  | Root | Raw |
|  |  | Unknown |
|  | Whole plant | Raw |
|  | Young leaf | Raw |
| *Capparis spinosa* L. | Floral bud | Preserved in brine |
|  |  | Preserved in salt |
|  |  | Preserved in vinegar |
|  | Flower | Raw |
|  | Fruit | Condiment |
|  |  | Preserved in brine |
|  |  | Preserved in salt |
|  |  | Preserved in vinegar |
|  |  | Unknown |
|  | Unknown | Unknown |
| *Capsella bursa-pastoris* (L.) Medic. | Aerial part | Raw |
| *Carlina acanthifolia* All. subsp. *cynara* (Pourr. ex Duby) Arcang. | Inflorescence | Boiled in milk |
|  |  | Boiled in water |
|  |  | Preserved in vinegar |
|  |  | Raw |
|  |  | Unknown |
|  | Unknown | Unknown |
| *Carlina acaulis* L. subsp. *caulescens* (Lam.) Schübl et Martens | Flower | Curd |
|  | Inflorescence | Raw |
| *Carum carvi* L. | Aerial part | Condiment |
|  | Fruit | Boiled in water |
|  |  | Condiment |
|  |  | Cooked in oil |
|  |  | Raw |
|  | Ripe fruit | Condiment |
| *Castanea sativa* Mill. | Fruit | Boiled in water |
|  |  | Cooked in oil |
|  |  | Cooked in sugar |
|  |  | Raw |
|  |  | Toasted |
|  |  | Unknown |
|  | Seed | Raw |
|  |  | Unknown |
| *Caucalis platycarpos* L. | Fruit | Toasted |
| *Celtis australis* L. | Fruit | Raw |
|  |  | Unknown |
|  | Ripe fruit | Raw |
| *Centaurea aspera* L. | Aerial part | Boiled in water |
|  |  | Unknown |
|  | Inflorescence | Boiled in water |
|  |  | Cooked in oil |
| *Centaurea scabiosa* L. | Aerial part | Raw |
| *Chaerophyllum hirsutum* L. | Leaf | Condiment |
| *Chamaemelum nobile* (L.) All. | Flowering top | Boiled in water |
|  |  | Condiment |
| *Chamaerops humilis* L. | Fruit | Raw |
|  | Leaf | Raw |
|  | Leafstalk | Raw |
|  | Ripe fruit | Raw |
|  | Stem | Raw |
|  | Unknown | Raw |
|  | Young shoot | Raw |
| *Chenopodium album* L. | Aerial part | Boiled in water |
|  | Leaf | Boiled in water |
| *Chenopodium ambrosioides* L. | Aerial part | Boiled in milk |
|  |  | Boiled in water |
|  | Leaf | Boiled in water |
| *Chenopodium bonus-henricus* L. | Aerial part | Boiled in water |
|  | Leaf | Boiled in milk |
|  |  | Boiled in water |
|  |  | Cooked in oil |
|  |  | Raw |
|  |  | Unknown |
|  | Unknown | Cooked in oil |
|  |  | Unknown |
|  | Young leaf | Raw |
| *Chondrilla juncea* L. | Aerial part | Raw |
|  | Leaf | Condiment |
|  |  | Raw |
|  | Unknown | Raw |
|  | Whole plant | Raw |
|  | Young aerial part | Boiled in water |
|  |  | Raw |
|  | Young leaf | Boiled in water |
|  |  | Cooked in oil |
|  |  | Preserved in vinegar |
|  |  | Raw |
|  |  | Unknown |
| *Cichorium endivia* L. subsp. *pumilum* (Jacq.) Cout. | Leaf | Cooked in oil |
|  |  | Raw |
| *Cichorium intybus* L. | Aerial part | Raw |
|  |  | Unknown |
|  | Inflorescence | Boiled in water |
|  | Leaf | Boiled in water |
|  |  | Boiled in water and oil |
|  |  | Cooked in oil |
|  |  | Raw |
|  |  | Toasted |
|  |  | Unknown |
|  | Root | Unknown |
|  | Seed | Boiled in water |
|  | Unknown | Boiled in water |
|  |  | Raw |
|  |  | Unknown |
|  | Young aerial part | Boiled in water and oil |
|  |  | Raw |
|  | Young leaf | Raw |
| *Cirsium arvense* (L.) Scop. | Aerial part | Boiled in water |
| *Cirsium echinatum* (Desf.) DC. in Lam. et DC. | Flower | Curd |
| *Clematis vitalba* L. | Fruit | Unknown |
| *Conopodium majus* (Gouan) Loret in Loret et Barr. | Root | Raw |
| *Convolvulus arvensis* L. | Aerial part | Raw |
|  | Flower | Raw |
|  | Leaf | Raw |
| *Convolvulus* sp. | Leaf | Raw |
| *Coriandrum sativum* L. | Fruit | Condiment |
|  | Leaf | Condiment |
|  |  | Raw |
|  | Unknown | Condiment |
| *Coriaria myrtifolia* L. | Fruit | Raw |
| *Coronilla minima* L. | Stem | Unknown |
| *Corylus avellana* L. | Aerial part | Raw |
|  | Dried fruit | Air dried |
|  |  | Raw |
|  |  | Unknown |
|  | Fruit | Air dried |
|  |  | Cooked in sugar |
|  |  | Raw |
|  |  | Toasted |
|  | Kernel | Toasted |
|  | Ripe fruit | Boiled in water |
|  | Seed | Air dried |
|  |  | Raw |
| *Crataegus monogyna* Jacq. | Flower | Cooked in oil |
|  | Fruit | Raw |
|  |  | Unknown |
|  | Unknown | Unknown |
| *Crepis vesicaria* L. | Leaf | Raw |
|  | Young leaf | Raw |
| *Crithmum maritimum* L. | Aerial part | Preserved in salt |
|  |  | Preserved in vinegar |
|  |  | Raw |
|  |  | Unknown |
|  | Leaf | Preserved in vinegar |
|  |  | Raw |
|  | Stem | Preserved in vinegar |
|  | Unknown | Preserved in vinegar |
|  | Young aerial part | Preserved in brine |
|  |  | Preserved in vinegar |
|  | Young leaf | Raw |
| *Cynara cardunculus* L. | Aerial part | Curd |
|  | Flower | Curd |
|  | Leaf | Curd |
|  | Leafstalk | Boiled in water |
|  |  | Condiment |
|  |  | Preserved in brine |
|  | Pappus | Curd |
|  | Stem | Boiled in water |
|  |  | Raw |
|  |  | Unknown |
|  | Stem with leaves / branches | Boiled in water |
|  | Unknown | Curd |
|  |  | Unknown |
| *Cyperus rotundus* L. | Rizome | Raw |
| *Cytinus hypocistis* (L.) L. | Aerial part | Raw |
| *Daucus carota* L. subsp. *carota* | Root | Unknown |
| *Delphinium staphisagria* L. | Unknown | Unknown |
| *Diplotaxis erucoides* (L.) DC. | Flower | Raw |
|  | Flower and leaf | Condiment |
|  | Fructified aerial part | Boiled in water |
|  | Inflorescence | Condiment |
| *Erodium malacoides* (L.) L'Hér. subsp. *malacoides* | Young leaf | Cooked in oil |
|  |  | Raw |
| *Eruca vesicaria* (L.) Cav. | Aerial part | Raw |
|  | Leaf | Raw |
|  | Young aerial part | Raw |
|  | Young leaf | Raw |
| *Erucastrum nasturtiifolium* (Poiret) O.E.Schulz | Aerial part | Unknown |
| *Eryngium campestre* L. | Stem | Raw |
|  | Young leaf | Raw |
|  | Young shoot | Raw |
| *Euphorbia* sp. | Latex | Curd |
| *Fagus sylvatica* L. | Fruit | Raw |
| *Ficus carica* L. | Aerial part | Raw |
|  | Infructescence | Air dried |
|  |  | Condiment |
|  |  | Cooked in sugar |
|  |  | Raw |
|  |  | Toasted |
|  |  | Unknown |
|  | Latex | Condiment |
|  |  | Curd |
|  |  | Unknown |
|  | Leaf | Condiment |
|  |  | Toasted |
|  |  | Unknown |
|  | Ripe fruit | Air dried |
|  |  | Boiled in water |
|  |  | Cooked in sugar |
|  |  | Raw |
|  |  | Toasted |
|  | Stem | Condiment |
|  |  | Curd |
|  | Unknown | Boiled in milk |
|  |  | Condiment |
|  | Young aerial part | Condiment |
|  | Young shoot | Curd |
| *Foeniculum vulgare* Mill. subsp. *piperitum* (Ucria) Cout. | Aerial part | Boiled in water |
|  |  | Boiled in water and fat |
|  |  | Condiment |
|  |  | Cooked in oil |
|  |  | Preserved in oil |
|  |  | Raw |
|  |  | Unknown |
|  | Bulb | Boiled in water |
|  |  | Condiment |
|  |  | Raw |
|  |  | Unknown |
|  | Flower | Cooked in oil |
|  | Flowering aerial part | Condiment |
|  | Fructified aerial part | Condiment |
|  | Fruit | Air dried |
|  |  | Condiment |
|  |  | Unknown |
|  | Inflorescence | Condiment |
|  |  | Raw |
|  | Leaf | Boiled in water |
|  |  | Boiled in water and fat |
|  |  | Condiment |
|  |  | Raw |
|  |  | Toasted |
|  |  | Unknown |
|  | Ripe fruit | Condiment |
|  | Root | Unknown |
|  | Seed | Condiment |
|  | Stem | Boiled in water |
|  |  | Condiment |
|  |  | Raw |
|  |  | Unknown |
|  | Stem with leaves / branches | Boiled in water |
|  |  | Preserved in brine |
|  |  | Unknown |
|  | Unknown | Boiled in water |
|  |  | Boiled in water and fat |
|  |  | Condiment |
|  |  | Preserved in brine |
|  |  | Raw |
|  |  | Unknown |
|  | Young aerial part | Boiled in water |
|  |  | Boiled in water and oil |
|  |  | Condiment |
|  |  | Cooked in oil |
|  |  | Raw |
|  | Young leaf | Boiled in water |
|  |  | Boiled in water and oil |
|  |  | Condiment |
|  |  | Cooked in oil |
| *Fragaria vesca* L. | Fruit | Cooked in sugar |
|  |  | Preserved in wine |
|  |  | Raw |
|  |  | Unknown |
|  | Immature fruit | Unknown |
|  | Infructescence | Cooked in sugar |
|  |  | Raw |
|  | Ripe fruit | Cooked in sugar |
|  |  | Raw |
|  |  | Unknown |
| *Fragaria viridis* Weston | Infructescence | Raw |
| *Fraxinus excelsior* L. | Bark | Unknown |
| *Gagea fistulosa* (Ram. ex DC.) Ker-Gawler | Bulb | Boiled in water |
| *Galactites tomentosa* Moench | Unknown | Raw |
| *Galium verum* L. | Unknown | Curd |
| *Gentiana lutea* L. | Rizome | Condiment |
|  | Root | Condiment |
|  |  | Unknown |
| *Globularia alypum* L. | Aerial part | Boiled in water |
|  |  | Condiment |
|  | Unknown | Boiled in water |
| *Glycyrrhiza glabra* L. | Rizome | Air dried |
|  |  | Condiment |
|  |  | Raw |
|  | Root | Raw |
|  |  | Unknown |
| *Helianthus tuberosus* L. | Leaf | Raw |
|  | Root | Boiled in water |
|  |  | Preserved in vinegar |
|  |  | Unknown |
|  | Tuber | Boiled in water |
|  |  | Boiled in water and oil |
|  |  | Preserved in high-grade alcohol |
|  |  | Preserved in vinegar |
|  |  | Preserved in wine |
|  |  | Raw |
|  |  | Unknown |
| *Helichrysum stoechas* (L.) Moench | Flowering top | Unknown |
|  | Leaf | Boiled in water |
|  |  | Condiment |
| *Humulus lupulus* L. | Aerial part | Condiment |
| *Hyoscyamus albus* L. | Leaf | Raw |
| *Hypericum perforatum* L. | Flower | Raw |
| *Hypochoeris radicata* L. | Aerial part | Raw |
| *Hyssopus officinalis* L. subsp. *canescens (*DC.) Nyman | Aerial part | Condiment |
|  | Flowering top | Condiment |
| *Inula graveolens* (L.) Desf. | Aerial part | Condiment |
| *Jasonia saxatilis* (Lam.) Guss. | Flowering aerial part | Boiled in water |
|  | Flowering top | Boiled in water |
| *Juglans regia* L. | Dried fruit | Air dried |
|  |  | Raw |
|  | Endocarp | Raw |
|  | Epidermis | Boiled in water |
|  | Fruit | Air dried |
|  |  | Condiment |
|  |  | Cooked in sugar |
|  |  | Raw |
|  |  | Unknown |
|  | Immature fruit | Raw |
|  |  | Toasted |
|  |  | Unknown |
|  | Ripe fruit | Preserved in vinegar |
|  |  | Raw |
|  | Seed | Air dried |
|  |  | Unknown |
| *Juniperus communis* L. | Cone | Cooked in sugar |
|  |  | Unknown |
|  | Fructification | Raw |
|  | Fruit | Air dried |
|  |  | Condiment |
|  |  | Cooked in sugar |
|  |  | Unknown |
|  | Ripe fruit | Boiled in water |
|  |  | Condiment |
|  |  | Cooked in sugar |
|  |  | Unknown |
|  | Unknown | Preserved in high-grade alcohol |
| *Juniperus oxycedrus* L. | Aerial part | Condiment |
|  | Cone | Raw |
|  |  | Unknown |
|  | Fructification | Condiment |
|  |  | Raw |
|  |  | Unknown |
|  | Seed | Condiment |
|  | Stem | Unknown |
|  | Unknown | Unknown |
| *Lactuca perennis* L. | Inflorescence | Raw |
|  | Leaf | Raw |
|  | Young leaf | Raw |
| *Lactuca serriola* L. | Aerial part | Unknown |
|  | Young aerial part | Raw |
|  |  | Unknown |
|  | Young leaf | Raw |
| *Lathyrus latifolius* L. | Immature fruit | Boiled in water |
| *Laurus nobilis* L. | Aerial part | Condiment |
|  | Fruit without seed | Condiment |
|  | Leaf | Air dried |
|  |  | Boiled in water and oil |
|  |  | Condiment |
|  |  | Cooked in oil |
|  |  | Preserved in oil |
|  |  | Preserved in vinegar |
|  |  | Unknown |
|  | Stem | Raw |
|  | Unknown | Unknown |
| *Lavandula latifolia* Medic. | Unknown | Boiled in water |
|  |  | Condiment |
| *Lavandula stoechas* L. subsp. *stoechas* | Aerial part | Condiment |
| *Lavatera arborea* L. | Flower | Cooked in oil |
|  |  | Raw |
| *Lavatera cretica* L. | Fruit | Raw |
|  |  | Unknown |
|  | Leaf | Toasted |
| *Leontodon hispidus* L. | Leaf | Raw |
| *Leontodon taraxacoides* (Vill.) Mérat | Unknown | Unknown |
| *Levisticum officinale* Koch | Leaf | Unknown |
| *Lolium rigidum* Gaud. | Aerial part | Raw |
| *Lonicera implexa* Ait. | Flower | Raw |
|  |  | Unknown |
| *Lonicera periclymenum* L. | Flower | Raw |
| *Lupinus albus* L. | Seed | Unknown |
|  | Young aerial part | Unknown |
| *Malva sylvestris* L. | Aerial part | Boiled in water |
|  | Flower | Raw |
|  | Fruit | Raw |
|  | Leaf | Cooked in oil |
|  |  | Unknown |
|  | Unknown | Raw |
|  | Young leaf | Boiled in water |
|  |  | Raw |
|  | Young shoot | Raw |
| *Mantisalca salmantica* (L.) Briq. et Cavill. | Leaf | Boiled in water |
|  |  | Boiled in water and oil |
|  | Young leaf | Raw |
| *Matricaria recutita* L. | Inflorescence | Unknown |
| *Medicago sativa* L. | Aerial part | Raw |
|  |  | Unknown |
|  | Flowering aerial part | Cooked in oil |
|  | Leaf | Boiled in water |
|  | Unknown | Raw |
|  | Whole plant | Unknown |
|  | Young shoot | Cooked in oil |
|  |  | Raw |
| *Melissa officinalis* L.subsp. *officinalis* | Aerial part | Condiment |
|  | Flowering top | Condiment |
|  | Leaf | Condiment |
|  |  | Unknown |
|  | Unknown | Condiment |
|  | Young aerial part | Condiment |
| *Mentha aquatica* L. | Aerial part | Boiled in water and oil |
|  |  | Condiment |
|  | Leaf | Condiment |
|  |  | Cooked in sugar |
|  | Whole plant | Condiment |
| *Mentha longifolia* (L.) Huds. | Aerial part | Condiment |
| *Mentha pulegium* L. | Aerial part | Boiled in water |
|  |  | Boiled in water and oil |
|  |  | Condiment |
|  |  | Preserved in high-grade alcohol |
|  | Flowering aerial part | Preserved in high-grade alcohol |
|  | Leaf | Boiled in water |
|  |  | Condiment |
|  | Whole plant | Condiment |
|  | Young aerial part | Cooked in sugar |
| *Mentha* sp. | Aerial part | Boiled in water and oil |
|  |  | Condiment |
|  |  | Unknown |
|  | Leaf | Boiled in water |
|  |  | Condiment |
|  |  | Raw |
|  | Young aerial part | Condiment |
| *Mentha spicata* L. | Aerial part | Boiled in water |
|  |  | Condiment |
|  |  | Unknown |
|  | Flowering top | Air dried |
|  |  | Boiled in water |
|  |  | Condiment |
|  | Leaf | Boiled in water |
|  |  | Condiment |
|  |  | Raw |
|  |  | Unknown |
|  | Unknown | Condiment |
|  |  | Unknown |
|  | Young leaf | Condiment |
| *Mentha suaveolens* Ehrh. | Flowering top | Condiment |
|  | Leaf | Condiment |
| *Mesembryanthemum crystallinum* L. | Aerial part | Condiment |
|  |  | Raw |
|  |  | Unknown |
| *Molopospermum peloponnesiacum* (L.) Koch | Aerial part | Raw |
|  | Defolied stem | Raw |
|  | Fruit | Cooked in sugar |
|  | Leaf | Raw |
|  | Stem | Cooked in sugar |
|  |  | Raw |
|  | Unknown | Raw |
|  |  | Unknown |
|  | Young leaf | Raw |
|  | Young shoot | Raw |
| *Montia fontana* L. | Aerial part | Raw |
| *Muscari comosum* (L.) Mill. | Bulb | Boiled in water and oil |
| *Muscari neglectum* Guss. ex Ten. | Bulb | Boiled in water and oil |
| *Myrtus communis* L. | Fruit | Cooked in sugar |
|  |  | Raw |
|  |  | Unknown |
|  |  | Cooked in sugar |
| *Nepeta cataria* L. | Unknown | Condiment |
| *Oenothera biennis* L. | Flower | Raw |
|  | Root | Unknown |
| *Onobrychis viciifolia* Scop. | Aerial part | Air dried |
| *Ononis tridentata* L. | Bark | Raw |
| *Opuntia maxima* Mill. | Cladode | Boiled in water |
|  | Fruit | Cooked in sugar |
|  |  | Raw |
|  | Leaf | Condiment |
| *Origanum virens* Hoffms. et Link | Aerial part | Condiment |
| *Origanum vulgare* L. | Aerial part | Condiment |
|  |  | Raw |
|  | Flowering aerial part | Condiment |
|  |  | Unknown |
|  | Flowering top | Condiment |
|  |  | Unknown |
|  | Inflorescence | Condiment |
|  | Leaf | Condiment |
|  |  | Raw |
|  | Stem with leaves / branches | Condiment |
|  | Unknown | Condiment |
|  |  | Unknown |
| *Oxalis acetosella* L. | Leaf | Raw |
|  |  | Unknown |
| *Oxalis corniculata* L. | Leaf | Raw |
| *Oxalis debilis* Humb., Bonpl. et Kunth. | Unknown | Raw |
| *Oxalis pes-caprae* L. | Floral leafstalk | Raw |
|  | Flower | Raw |
|  | Stem | Raw |
|  |  | Unknown |
|  | Unknown | Raw |
| *Panicum miliaceum* L. | Seed | Air dried |
| *Papaver dubium* L. | Leaf | Raw |
| *Papaver rhoeas* L. | Aerial part | Condiment |
|  |  | Raw |
|  | Leaf | Boiled in water |
|  |  | Cooked in oil |
|  |  | Raw |
|  | Seed | Condiment |
|  | Unknown | Raw |
|  | Young aerial part | Raw |
|  |  | Unknown |
|  | Young leaf | Raw |
| *Papaver somniferum* L. | Seed | Unknown |
| *Parietaria officinalis* L. subsp. *judaica* (L.) Béguinot | Aerial part | Boiled in water |
|  |  | Raw |
| *Physalis alkekengi* L. | Fruit | Raw |
| *Picris echioides* L. | Leaf | Boiled in water |
|  |  | Unknown |
| *Pinus halepensis* Mill. | Cone | Unknown |
| *Pinus pinea* L. | Cone | Raw |
|  | Dried fruit | Air dried |
|  | Fruit | Air dried |
|  |  | Condiment |
|  |  | Raw |
|  |  | Toasted |
|  |  | Unknown |
|  | Seed | Air dried |
|  |  | Raw |
|  |  | Toasted |
|  |  | Unknown |
| *Pinus* sp. | Young aerial part | Condiment |
| *Pistacia lentiscus* L. | Fruit | Unknown |
|  | Leaf | Air dried |
|  |  | Unknown |
| *Plantago coronopus* L. subsp. *coronopus* | Young aerial part | Raw |
| *Plantago lanceolata* L. | Leaf | Boiled in water |
|  |  | Cooked in oil |
|  |  | Raw |
|  |  | Unknown |
|  | Seed | Raw |
|  | Whole plant | Raw |
|  | Young leaf | Raw |
| *Plantago major* L. subsp. *major* | Leaf | Boiled in water |
|  |  | Condiment |
|  |  | Raw |
|  | Young leaf | Raw |
| *Polygala calcarea* F.W.Schultz | Flower | Raw |
| *Polygonatum odoratum* (Mill.) Druce | Unknown | Unknown |
| *Polygonum aviculare* L. | Aerial part | Raw |
| *Portulaca oleracea* L. | Aerial part | Boiled in water |
|  |  | Boiled in water and oil |
|  |  | Raw |
|  | Flower | Raw |
|  | Leaf | Boiled in water |
|  |  | Cooked in oil |
|  |  | Raw |
|  |  | Toasted |
|  |  | Unknown |
|  | Unknown | Condiment |
|  |  | Raw |
|  |  | Unknown |
|  | Young aerial part | Boiled in water |
|  |  | Cooked in oil |
|  |  | Raw |
|  |  | Unknown |
|  | Young leaf | Preserved in vinegar |
|  |  | Raw |
|  | Young shoot | Raw |
| *Prunella grandiflora* (L.) Scholler | Flower | Raw |
| *Prunus avium* L. subsp. *sylvestris* (Kirschl.) Dierb. | Fruit | Cooked in sugar |
|  | Ripe fruit | Cooked in sugar |
| *Prunus spinosa* L. | Fructified aerial part | Toasted |
|  | Fruit | Condiment |
|  |  | Cooked in sugar |
|  |  | Preserved in high-grade alcohol |
|  |  | Raw |
|  |  | Unknown |
|  | Ripe fruit | Cooked in sugar |
|  |  | Raw |
|  |  | Unknown |
| *Pteridium aquilinum* (L.) Kuhn | Frond | Raw |
| *Pulmonaria affinis* Jord. in F.W.Schultz | Flower | Raw |
| *Punica granatum* L. | Fruit | Cooked in sugar |
|  |  | Raw |
|  |  | Unknown |
|  | Infructescence | Raw |
|  | Ripe fruit | Raw |
|  |  | Unknown |
|  | Seed | Cooked in sugar |
|  |  | Unknown |
| *Pyrus communis* L. subsp. *pyraster* (L.) Asch. et Graebn. | Ripe fruit | Raw |
| *Pyrus malus* L. subsp. *sylvestris* (L.) Ehrh. | Ripe fruit | Raw |
| *Pyrus spinosa* Forsk. | Fruit | Raw |
| *Quercus coccifera* L. | Fruit | Toasted |
| *Quercus ilex* L. | Bark | Raw |
|  | Dried fruit | Air dried |
|  | Fruit | Boiled in water |
|  |  | Raw |
|  |  | Toasted |
|  |  | Unknown |
|  | Seed | Air dried |
|  |  | Unknown |
| *Quercus pubescens* Willd. | Ripe fruit | Air dried |
| *Quercus suber* L. | Fruit | Air dried |
| *Reichardia picroides* (L.) Roth | Aerial part | Condiment |
|  |  | Raw |
|  | Leaf | Cooked in oil |
|  |  | Raw |
|  |  | Unknown |
|  | Unknown | Raw |
|  |  | Unknown |
|  | Young aerial part | Raw |
|  | Young leaf | Boiled in water |
|  |  | Raw |
|  |  | Unknown |
| *Ribes alpinum* L. | Fruit | Cooked in sugar |
|  | Ripe fruit | Cooked in sugar |
|  |  | Raw |
| *Ribes nigrum* L. | Fruit | Cooked in sugar |
|  |  | Raw |
| *Ribes petraeum* Wulfen in Jacq. | Fruit | Cooked in sugar |
|  |  | Raw |
| *Ribes rubrum* L. | Ripe fruit | Unknown |
| *Ribes uva-crispa* L. | Fruit | Raw |
| *Robinia pseudoacacia* L. | Flower | Cooked in oil |
|  |  | Raw |
| *Rorippa nasturtium-aquaticum* (L.) Hayek subsp. *nasturtium-aquaticum* | Aerial part | Boiled in water |
|  |  | Cooked in oil |
|  |  | Raw |
|  |  | Unknown |
|  | Leaf | Cooked in oil |
|  |  | Raw |
|  | Stem | Boiled in water |
|  | Unknown | Raw |
|  | Whole plant | Raw |
|  | Young aerial part | Boiled in water |
|  |  | Preserved in vinegar |
|  |  | Raw |
|  |  | Unknown |
|  | Young leaf | Cooked in oil |
|  |  | Raw |
| *Rosa agrestis* Savi | Corolla | Cooked in sugar |
|  | Fruit | Cooked in sugar |
|  |  | Raw |
|  | Ripe fruit | Cooked in sugar |
| *Rosa canina* L. | Corolla | Cooked in sugar |
|  | Fruit | Cooked in sugar |
|  |  | Raw |
|  | Leaf | Condiment |
|  | Ripe fruit | Cooked in sugar |
| *Rosa micrantha* Borrer ex Sm. in Sowerby | Corolla | Cooked in sugar |
|  | Ripe fruit | Cooked in sugar |
| *Rosa pimpinellifolia* L. subsp. *pimpinellifolia* | Fruit | Boiled in water |
|  |  | Cooked in oil |
|  |  | Cooked in sugar |
|  | Young aerial part | Raw |
| *Rosa pouzinii* Tratt. | Corolla | Cooked in sugar |
|  | Ripe fruit | Cooked in sugar |
| *Rosa* sp. | Corolla | Cooked in sugar |
|  |  | Raw |
|  | Flower | Condiment |
|  |  | Raw |
|  | Fruit | Cooked in sugar |
|  | Unknown | Cooked in sugar |
| *Rosa tomentosa* Sm. | Fruit | Cooked in sugar |
|  |  | Raw |
| *Rosmarinus officinalis* L. | Aerial part | Condiment |
|  |  | Raw |
|  |  | Unknown |
|  | Flower | Raw |
|  | Flowering aerial part | Condiment |
|  |  | Unknown |
|  | Flowering stem | Condiment |
|  | Flowering top | Condiment |
|  |  | Unknown |
|  | Inflorescence | Condiment |
|  | Leaf | Condiment |
|  | Stem with leaves / branches | Condiment |
|  | Unknown | Boiled in water |
|  |  | Condiment |
|  |  | Unknown |
|  | Young aerial part | Condiment |
| *Rubia peregrina* L. subsp. *peregrina* | Aerial part | Unknown |
| *Rubus caesius* L. | Ripe fruit | Raw |
| *Rubus idaeus* L. | Fruit | Cooked in sugar |
|  |  | Raw |
|  |  | Unknown |
|  | Infructescence | Condiment |
|  |  | Cooked in sugar |
|  |  | Raw |
|  | Ripe fruit | Cooked in sugar |
|  |  | Raw |
|  |  | Unknown |
|  | Unknown | Cooked in sugar |
| *Rubus ulmifolius* Schott | Fruit | Boiled in water |
|  |  | Condiment |
|  |  | Cooked in sugar |
|  |  | Raw |
|  |  | Unknown |
|  | Ripe fruit | Cooked in sugar |
|  |  | Raw |
|  | Stem | Raw |
|  |  | Unknown |
|  | Unknown | Unknown |
|  | Young aerial part | Raw |
|  | Young shoot | Cooked in oil |
|  |  | Raw |
|  |  | Unknown |
| *Rumex acetosa* L. | Aerial part | Raw |
|  | Leaf | Boiled in water |
|  |  | Raw |
| *Rumex acetosella* L. subsp. *angiocarpus* Murb. | Leaf | Raw |
|  | Young aerial part | Raw |
|  |  | Unknown |
| *Rumex crispus* L. | Leaf | Raw |
| *Rumex obtusifolius* L. | Aerial part | Unknown |
| *Rumex pulcher* L. | Aerial part | Unknown |
| *Rumex scutatus* L. | Leaf | Raw |
| *Rumex* sp. | Leaf | Raw |
|  | Unknown | Condiment |
| *Ruta chalepensis* L. | Aerial part | Air dried |
|  |  | Condiment |
|  |  | Raw |
|  |  | Unknown |
|  | Leaf | Condiment |
|  |  | Unknown |
|  | Unknown | Unknown |
| *Ruta graveolens* L. | Fulla | Condiment |
|  | Part aèria | Condiment |
|  |  | Unknown |
| *Salicornia patula* Duval-Jouve | Aerial part | Raw |
| *Salvia officinalis* L. subsp. *lavandulifolia* (Vahl) Gams | Aerial part | Condiment |
|  | Flowering top | Condiment |
|  | Leaf | Condiment |
|  | Unknown | Condiment |
| *Salvia verbenaca* L. | Unknown | Unknown |
| *Sambucus nigra* L. | Flowering aerial part | Cooked in oil |
|  | Fruit | Boiled in water |
|  |  | Cooked in sugar |
|  |  | Cooked with wine |
|  |  | Raw |
|  | Inflorescence | Condiment |
|  |  | Cooked in oil |
|  |  | Cooked in sugar |
|  |  | Unknown |
|  | Infructescence | Condiment |
|  |  | Cooked in sugar |
|  | Ripe fruit | Boiled in water |
|  | Seed | Cooked in sugar |
| *Samolus valerandi* L. | Aerial part | Raw |
|  | Whole plant | Raw |
|  |  | Unknown |
| *Sanguisorba minor* Scop. | Aerial part | Unknown |
|  | Unknown | Raw |
|  | Young aerial part | Unknown |
| *Santolina chamaecyparissus* L. subsp. *magonica* O.Bolòs, Molinier et P.Monts. | Flowering top | Boiled in water |
|  |  | Condiment |
| *Santolina chamaecyparissus* L. subsp. *squarrosa* (DC.) Nyman | Unknown | Condiment |
| *Sarothamnus scoparius* (L.) Wimm. ex Koch | Unknown | Raw |
| *Satureja calamintha* (L.) Scheele | Flowering aerial part | Condiment |
|  |  | Unknown |
|  | Flowering top | Condiment |
|  | Inflorescence | Condiment |
|  | Leaf | Boiled in water |
|  | Stem with leaves / branches | Preserved in brine |
| *Satureja fruticosa* (L.) Briq. | Aerial part | Boiled in water and oil |
|  | Flowering aerial part | Condiment |
|  |  | Unknown |
| *Satureja hortensis* L. | Aerial part | Condiment |
|  | Flowering aerial part | Condiment |
|  | Flowering top | Condiment |
|  | Leaf | Condiment |
|  | Young aerial part | Condiment |
| *Satureja montana* L. | Aerial part | Condiment |
|  | Cortical parenchyma | Condiment |
|  | Flowering aerial part | Condiment |
|  | Flowering top | Condiment |
|  |  | Unknown |
|  | Inflorescence | Condiment |
|  | Leaf | Condiment |
|  | Stem with leaves / branches | Preserved in brine |
|  | Unknown | Condiment |
|  |  | Preserved in brine |
| *Satureja obovata* Lag. subsp. *gracilis* Willk. | Leaf | Condiment |
| *Saxifraga aquatica* Lap. | Leaf | Raw |
| *Scirpus holoschoenus* L. | Leaf | Raw |
|  |  | Unknown |
|  | Rizome | Raw |
|  | Root | Raw |
|  | Stem | Raw |
|  | Young aerial part | Raw |
| *Scolymus hispanicus* L. | Stem | Raw |
| *Scorzonera laciniata* L. | Young leaf | Raw |
| *Scrophularia auriculata* L. | Leaf | Raw |
| *Sedum acre* L. | Aerial part | Unknown |
| *Sedum rupestre* L. | Unknown | Unknown |
| *Sedum* sp. | Aerial part | Preserved in brine |
|  | Unknown | Unknown |
| *Sideritis hyssopifolia* L. | Flowering top | Boiled in water |
|  |  | Condiment |
| *Silene vulgaris* (Moench) Garcke | Aerial part | Unknown |
|  | Flower | Condiment |
|  |  | Raw |
|  | Flowering top | Toasted |
|  | Leaf | Boiled in water |
|  |  | Boiled in water and fat |
|  |  | Boiled in water and oil |
|  |  | Condiment |
|  |  | Cooked in oil |
|  |  | Raw |
|  |  | Toasted |
|  |  | Unknown |
|  | Root | Cooked in oil |
|  |  | Preserved in vinegar |
|  | Stem | Cooked in oil |
|  |  | Raw |
|  | Unknown | Boiled in water |
|  |  | Cooked in oil |
|  |  | Unknown |
|  | Young aerial part | Boiled in water |
|  |  | Condiment |
|  |  | Raw |
|  | Young leaf | Boiled in water |
|  |  | Boiled in water and oil |
|  |  | Condiment |
|  |  | Cooked in oil |
|  |  | Raw |
| *Silybum marianum* (L.) Gaertn. | Flower | Curd |
|  | Inflorescence | Raw |
|  | Leaf | Boiled in water |
|  | Stem | Boiled in water |
| *Sonchus asper* (L.) Hill | Stem | Raw |
|  | Young leaf | Raw |
| *Sonchus oleraceus* L. | Aerial part | Raw |
|  | Leaf | Raw |
|  |  | Unknown |
|  | Unknown | Raw |
|  | Young aerial part | Raw |
|  | Young leaf | Raw |
| *Sonchus* sp. | Young aerial part | Raw |
| *Sonchus tenerrimus* L. | Aerial part | Raw |
|  | Leaf | Boiled in water |
|  |  | Raw |
|  | Young aerial part | Raw |
|  | Young leaf | Raw |
| *Sorbus domestica* L. | Fruit | Air dried |
|  |  | Raw |
|  |  | Unknown |
|  | Ripe fruit | Air dried |
|  |  | Boiled in water and oil |
|  |  | Cooked in sugar |
|  |  | Raw |
|  |  | Unknown |
| *Sorbus torminalis* (L.) Crantz | Fruit | Raw |
| *Sorghum bicolor* (L.) Moench | Seed | Toasted |
| *Stellaria media* (L.) Vill. | Aerial part | Raw |
| *Tanacetum balsamita* L. | Aerial part | Condiment |
|  | Leaf | Condiment |
|  | Unknown | Condiment |
| *Tanacetum parthenium* (L.) Schultz Bip. | Unknown | Condiment |
| *Taraxacum dissectum* (Ledeb.) Ledeb. | Leaf | Raw |
|  |  | Toasted |
|  | Unknown | Unknown |
|  | Young leaf | Raw |
| *Taraxacum officinale* Weber in Wiggers | Aerial part | Boiled in water |
|  |  | Raw |
|  | Inflorescence | Cooked in sugar |
|  |  | Raw |
|  | Leaf | Boiled in water |
|  |  | Cooked in oil |
|  |  | Raw |
|  | Root | Condiment |
|  | Unknown | Raw |
|  |  | Unknown |
|  | Whole plant | Raw |
|  | Young aerial part | Boiled in water |
|  |  | Raw |
|  | Young leaf | Raw |
|  |  | Unknown |
| *Taraxacum* sp. | Fruit | Cooked in sugar |
|  | Leaf | Raw |
| *Taxus baccata* L. | Aryl | Raw |
|  | Fruit | Raw |
| *Teucrium chamaedrys* L. | Aerial part | Condiment |
| *Teucrium polium* L. | Aerial part | Condiment |
|  | Flowering aerial part | Condiment |
|  | Unknown | Unknown |
| *Thymbra capitata* (L.) Cav. | Aerial part | Condiment |
|  | Flowering top | Condiment |
|  | Leaf | Condiment |
| *Thymus piperella* L. | Aerial part | Condiment |
|  | Fruit juice | Condiment |
|  | Leaf | Condiment |
| *Thymus serpyllum* L. | Aerial part | Boiled in water |
|  |  | Condiment |
|  | Flowering aerial part | Boiled in water |
|  |  | Condiment |
|  | Flowering top | Boiled in water |
|  |  | Condiment |
|  |  | Raw |
|  |  | Unknown |
|  | Inflorescence | Condiment |
|  |  | Unknown |
|  | Nectar | Condiment |
|  | Unknown | Boiled in water |
| *Thymus* sp. | Aerial part | Condiment |
|  | Flowering top | Condiment |
|  | Unknown | Condiment |
| *Thymus vulgaris* L. | Aerial part | Boiled in water |
|  |  | Boiled in water and oil |
|  |  | Condiment |
|  |  | Preserved in brine |
|  |  | Unknown |
|  | Cortical parenchyma | Boiled in water |
|  | Flower | Boiled in water |
|  | Flowering aerial part | Boiled in water |
|  |  | Condiment |
|  |  | Unknown |
|  | Flowering stem | Boiled in water |
|  |  | Condiment |
|  | Flowering top | Air dried |
|  |  | Boiled in water |
|  |  | Condiment |
|  |  | Unknown |
|  | Inflorescence | Boiled in water |
|  |  | Condiment |
|  |  | Cooked in oil |
|  |  | Raw |
|  | Leaf | Boiled in water |
|  |  | Condiment |
|  | Stem with leaves / branches | Boiled in water |
|  |  | Condiment |
|  |  | Unknown |
|  | Unknown | Boiled in water and oil |
|  |  | Condiment |
|  | Young aerial part | Condiment |
| *Tilia platyphyllos* Scop. | Flower and leaf | Boiled in water |
|  | Inflorescence | Boiled in water |
|  |  | Condiment |
|  | Inflorescence with bract | Boiled in water |
|  | Leaf | Boiled in water |
| *Tragopogon porrifolius* L. | Root | Cooked in oil |
|  |  | Raw |
| *Trifolium alpinum* L. | Bract | Raw |
|  | Leaf | Condiment |
|  | Root | Condiment |
|  |  | Raw |
| *Trifolium incarnatum* L. | Flowering aerial part | Boiled in water |
| *Ulmus minor* Mill. | Leaf | Boiled in water |
|  |  | Raw |
| *Urginea maritima* (L.) Baker | Bulb | Unknown |
| *Urospermum dalechampii* (L.) Scop. ex F.W.Schmidt | Leaf | Raw |
|  | Root | Raw |
|  | Young aerial part | Unknown |
| *Urtica dioica* L. | Aerial part | Boiled in water |
|  |  | Cooked in oil |
|  |  | Raw |
|  |  | Unknown |
|  | Flowering top | Condiment |
|  | Leaf | Boiled in water |
|  |  | Boiled in water and oil |
|  |  | Condiment |
|  |  | Cooked in oil |
|  |  | Unknown |
|  | Unknown | Boiled in water |
|  |  | Raw |
|  | Young aerial part | Boiled in water |
|  |  | Cooked in oil |
|  |  | Raw |
|  | Young leaf | Cooked in oil |
| *Urtica membranacea* Poiret in Lam. | Aerial part | Raw |
|  | Leaf | Boiled in water and oil |
|  | Young aerial part | Boiled in water |
|  |  | Boiled in water and oil |
| *Urtica* sp. | Aerial part | Boiled in water |
|  |  | Cooked in oil |
|  | Leaf | Boiled in water and fat |
|  |  | Boiled in water and oil |
|  |  | Condiment |
|  |  | Cooked in oil |
|  |  | Raw |
|  | Young aerial part | Boiled in water |
|  |  | Cooked in oil |
|  |  | Raw |
| *Urtica urens* L. | Fruit juice | Raw |
|  | Leaf | Boiled in water |
|  |  | Cooked in oil |
|  |  | Raw |
|  |  | Unknown |
|  | Young aerial part | Cooked in oil |
|  |  | Raw |
|  |  | Unknown |
| *Vaccinium myrtillus* L. | Fruit | Cooked in sugar |
|  |  | Raw |
|  |  | Unknown |
|  | Ripe fruit | Cooked in sugar |
|  | Unknown | Cooked in sugar |
| *Valerianella locusta* (L.) Laterrade | Aerial part | Raw |
|  | Leaf | Raw |
|  | Young leaf | Raw |
| *Valerianella* sp. | Aerial part | Raw |
| *Verbascum thapsus* L. | Seed | Unknown |
| *Veronica hederifolia* L. | Unknown | Condiment |
| *Vicia ervilia* (L.) Willd. | Stem | Unknown |
| *Vicia peregrina* L. | Aerial part | Raw |
| *Vicia sativa* L. | Aerial part | Raw |
|  | Seed | Boiled in water |
|  | Unknown | Raw |
| *Viola odorata* L. | Flower | Condiment |
| *Viola tricolor* L. | Flower | Raw |
| *Vitex agnus-castus* L. | Flowering aerial part | Raw |
