# Supplementary material for: The Power of Wild Plants in Feeding Humanity: A Meta-Analytic Ethnobotanical Approach in the Catalan Linguistic Area
Source: Foods. 2020 Dec 29;10(1):61. doi: 10.3390/foods10010061 (PMC7824323; doi:10.3390/foods10010061)
Supplement: Supplementary file 1 [file foods-10-00061-s001.zip › Supplementary material 1.docx]

**Supplementary material 1.** Studied areas and research works analysed in the present work

| **Studied areas** | **References** |
| --- | --- |
| Alt Empordà (Catalonia) and a part of Vallespir (Northern Catalonia) | Parada, M. Estudi etnobotànic de l’Alt Empordà; PhD thesis, Universitat de Barcelona, 2007. |
| Anoia (Catalonia) | Talavera, M. La recuperació dels coneixements tradicionals relatius a la biodiversitat com a eina per al desenvolupament d'un model agroalimentari més sostenible: estudi etnobotànic de la comarca de l'Anoia, desenvolupament de nous cultius amb espècies silvestres, i acceptació per part dels consumidors dels productes elaborats amb espècies silvestres i varietats tradicionals; PhD thesis, Universitat de Barcelona, 2018. |
| Baix Llobregat (Catalonia) | Marín (unpubl. res.) |
| Castelló (Valencia) | Mulet, L. Aportaciones al conocimiento etnobotánico de la provincia de Castellón; PhD thesis, Universitat de València, 1990. |
| Cerdanya (Catalonia and Northern Catalonia), Capcir, Conflent (Northern Catalonia) | Muntané, J. Aportació al coneixement de l’etnobotànica de Cerdanya; PhD thesis, Univer­sitat de Barcelona, 1991.  Muntané, J. Etnobotànica, etnofarmàcia i tradicions po­pulars de la Catalunya septentrional (Capcir, Cerdanya i Conflent); PhD thesis, Universitat de Barcelona, 2005. |
| Formentera (Balearic Islands) | Mayans, M. Estudi etnobotànic de Formentera; Master thesis, Universitat de Barcelona, 2013. |
| Gallecs (Catalonia) | Bonet, M.À.; Roldán, M.; Camprubí, J.; Vallès, J. *Etnobotànica de Gallecs. Plantes i cultura popular al Baix Vallès*; Centre d’Estudis Molletans: Mollet del Vallès, Catalonia, Spain, 2008. |
| Garrigues and a part of Segrià (Catalonia) | Gras, A. Dades etnobotàniques de Catalunya: metanàlisi i bioprospecció; PhD thesis, Universitat de Barcelona, 2019. |
| Gavarres (Catalonia) | Blanch, A. Estudi etnobotànic de la regió geogràfica del Massís de les Gavarres (província de Girona); Undergraduate work, Universitat de Barcelona, 2013.  Saura, S. *Usos i cultura popular de les plantes a les Gavarres*; Consorci de les Gavarres: Monells, Catalonia, Spain, 2009. |
| Gironès (Catalonia) | Pagès, L. Estudi etnobotànic al municipi de Cassà de la Selva; Degree thesis, Universitat de Barcelona, 2013.  Serrasolses, G. Estudis etnobotànics del Gironès occidental; Master thesis, Universitat de Barcelona, 2014. |
| Guilleries (Catalonia) | Selga, A. Estudis etnobotànics a les Guilleries. Universitat de Barcelona; Master thesis. 1998. |
| Mallorca (Balearic Islands) | Carrió, E. Contribució a l’etnobotànica de Mallorca. La biodiversitat vegetal i la seva gestió en una illa mediterrània; PhD thesis, Universitat de Barcelona, 2013. |
| Montseny (Catalonia) | Bonet, M.À. Estudi etnobotànic del Montseny; PhD thesis, Universitat de Barcelona, 2001. |
| Pallars Sobirà, Pallars Jussà (Catalonia) | Agelet, A. Estudis d’etnobotànica farmacèutica al Pallars; PhD thesis, Universitat de Barcelona, 1999. |
| Pla de l’Estany (Catalonia) | Planas, I. L’ús de les plantes al Pla de l’Estany; Master work, Universitat de Barcelona, 2011.  Sala (unpubl. res.) |
| Prades mountains (Catalonia) | Batet, D.; Cartanyà. J.; Castells. R.; Piñas, I.; Salat, X. *Etnobotànica a les muntanyes de Prades*; Centre d’Història Natural de la Conca de Barberà: Montblanc, Catalonia, Spain, 2011. |
| Ripollès (Catalonia) | Díaz, E. Iniciació a la recerca etnobotànica. Estudi etnobotànic del Ripollès; Undergraduate work, Universitat de Barcelona, 2009.  Rigat, M.; Gras, A.; Vallès, J.; Garnatje, T. Estudis etnobotànics a la comarca del Ripollès (Pirineu, Catalunya, península Ibèrica). *Collect. Bot.* **2017**, *36*, e003. doi: 10.3989/collectbot.2017.v36.003 |
| Segarra (Catalonia) | Raja D. Estudis etnobotànics a la comarca de la Segarra; Degree thesis, Universitat de Barcelona, 1995. |
| Mariola mountain range (Valencia) | Santamaria, M. Mariola: gents, culte i plantes medicinals. Una petita aventura etnobotànica; Undergraduate work, Universitat de Barcelona, 2007. |
| Simat de la Valldigna (Valencia) | Ferrando, F. Estudi etnobotànic a Simat de la Valldigna (la Safor, País Valencià); Master thesis, Universitat de Barcelona, 2012. |
| Ulldemolins (Catalonia) | Llurba, N. Estudi etnobotànic d’Ulldemolins (Priorat); Undergraduate work, Universitat de Barcelona, 2009. |
| Val d’Aran (Catalonia) | Aldea, C.; Almeida, B.; Garnatje, T.; Vallès, J. *Estudi etnobotànic ena Val d’Aran. Sabença populara e patrimoni naturau e culturau*; Edicions de la Universitat de Barcelona: Barcelona, Catalonia, Spain, 2019. |
| Vall d’en Bas (Catalonia) | Plana, E. Estudi etnobotànic de la Vall d’en Bas; Undergraduate work, Universitat de Barcelona, 2013. |
| Vall del Cardener (Catalonia) | Cano, M. Estudi etnobotànic al Bages. L’ús tradicional de les plantes de la vall del Cardener; High school work, Manresa High School, 2016. |
| Vall del Tenes (Catalonia) | Bonet, M.À. Estudis etnobotànics a la vall del Tenes (Vallès Oriental); Degree thesis, Universitat de Barcelona, 1991. |
| Vallès Occidental (Catalonia) | Nogueras, A. Estudi etnobotànic a Martorelles, Santa Maria de Martorelles i Sant Fost de Campsentelles (Vallès Oriental, Catalunya); Degree thesis, Universitat de Barcelona, 2013. |
